# Tuliposides H–J and Bioactive Components from the Bulb of *Amana edulis*

**DOI:** 10.3390/molecules26195907

**Published:** 2021-09-29

**Authors:** Chia-Lin Lee, Zhi-An Gao, Yun-Lian Jhan, Yuan-Shiun Chang, Chao-Jung Chen

**Affiliations:** 1 Department of Cosmeceutics, China Medical University, Taichung 406040, Taiwan; s10061131@gmail.com (Z.-A.G.); u9884002@cmu.edu.tw (Y.-L.J.); 2 Chinese Medicine Research and Development Center, China Medical University Hospital, Taichung 40402, Taiwan; 3 Chinese Medicine Research Center, China Medical University, Taichung 40402, Taiwan; 4 Department of Chinese Pharmaceutical Sciences and Chinese Medicine Resources, China Medical University, Taichung 40402, Taiwan; yschang@mail.cmu.edu.tw; 5 Graduate Institute of Integrated Medicine, China Medical University, Taichung 40402, Taiwan; ironmanchen@yahoo.com.tw; 6 Proteomics Core Laboratory, Department of Medical Research, China Medical University Hospital, Taichung 40402, Taiwan

**Keywords:** *Amana edulis*, Liliaceae, bulb, tuliposides H–J, melanogenesis regulation

## Abstract

Three new tuliposides H–J (**1**–**3**) and 11 known compounds were obtained from the methanolic extracts of the bulbs of *Amana edulis* for the first time. Their structures were elucidated by NMR, MS, and IR spectroscopic data, optical rotation, and Mosher’s method. The melanogenesis properties of all the isolates were evaluated in B16 melanoma cells. Consequently, tributyl citrate (**9**) had anti-melanogenesis activity but was cytotoxic toward B16. (+)-Pyroglutamic acid (**4**), (+)-butyl 5-oxopyrrolidine-2-carboxylate (**6**), (–)-3-hydroxy-2-methylbutyrolactone (**10**), and 5-(hydroxymethyl)furfural (**12**) had increased melanin productions and tyrosinase activities. Those active components could be further studied as the candidates against melanoma and vitiligo for skin diseases or whitening/hypopigmentation for hair.

## 1. Introduction

**Figure 1:**
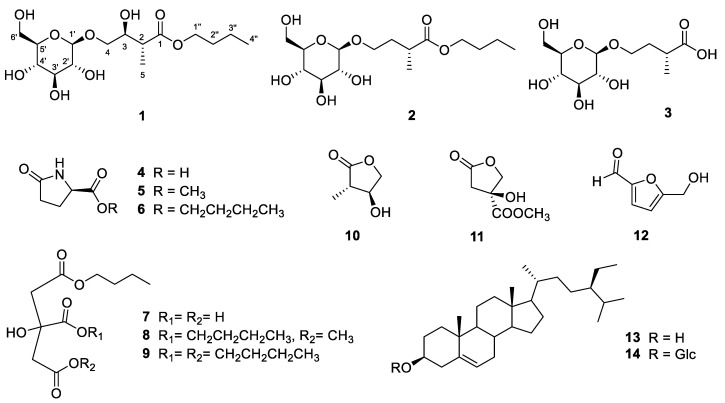
Structures of compounds **1**−**14**.

*Amana edulis* (*A. edulis*; Miq.) Honda, syn. *Tulipa edulis* (Miq.) Baker belongs to the Liliaceae family and is a folk medicinal plant used to treat cancer diseases [1,2]. However, there have been few phytochemical and biological studies of this species reported to date. For example, 95% and 50% EtOH extracts could induce apoptosis in human gastric (SGC-7901) [1] and hepatoma (BEL7404, HepG2, and Huh7) [2] carcinoma cells, respectively. The polysaccharides prepared from *A. edulis* have anti-oxidant properties, including DPPH, OH^−^, and ABTS^+^ scavenging activities [3,4,5]. In a research of new agents from natural products for skin disorders, we found that the MeOH extracts of the bulbs of *A. edulis* show potential melanogenesis regulation that has not yet been researched. When exposed to the ultraviolet radiation of solar light or harmful chemicals and pathogenic factors, moderate melanogenesis can protect our skin from reactive oxygen species generation in keratinocytes and melanocytes [6]. Tyrosinase is an important enzyme in melanogenesis to produce more melanin for us to defense against aforementioned hazardous conditions [7]. Therefore, the active components of *A. edulis* are worthy to be clarified for further medical applications. In this investigation, three new tuliposides H–J (**1**–**3**) and 11 known compounds (**4**–**14**) (Figure 1) were isolated from *A. edulis* and structurally identified by NMR, MS, and IR spectroscopic data, optical rotation, or Mosher ester reaction. Compound **9** could decrease melanin contents but be cytotoxic toward B16 melanoma cells. Compounds **4**, **6**, **10**, and **12** had increased melanin productions and tyrosinase activities. Overall, this work proved *A. edulis* and its active components could be new agents for melanin-related diseases, such as melanoma and hypopigmentation (skin vitiligo or hair whitening).

## 2. Results and Discussion

### 2.1. Structure Elucidations of Isolates **1**–**3**

The MeOH extract (TEM) of the bulbs of *A. edulis* was partitioned into EtOAc-soluble (TEE), *n*-BuOH-soluble (TEB), and H_2_O-soluble (TEW) fractions separately. The fractionation of the TEE and TEB extracts afforded new isoprenoid glycosides **1**–**3** and known compounds **4**–**14** (Figure 1). Additionally, the major components of the TEW extract were carbohydrates.

**Figure 2:**
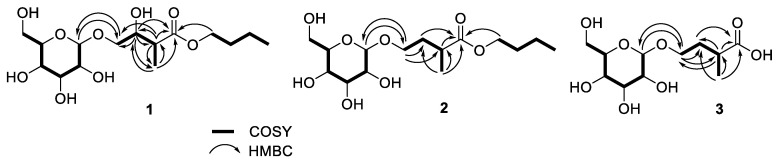
Key COSY and HMBC correlations of compounds **1**–**3**.

**Figure 3:**
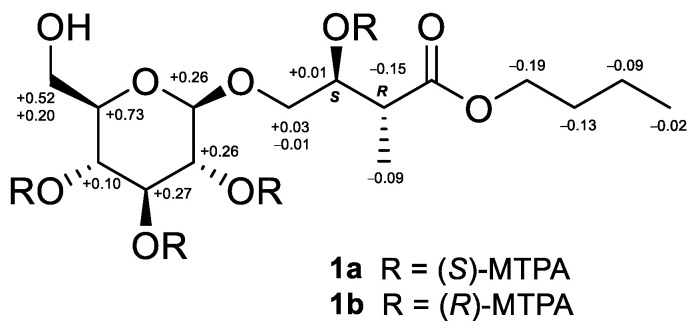
^1^H NMR chemical shift differences [*δ* (*S*)-MTPA—*δ* (*R*)-MTPA] of the MTPA esters **1a** and **1b**.

The HRESIMS (high-resolution electrospray ionization mass spectrometry) of **1** showed a [M − H]^−^ ion at *m*/*z* 351.1652, indicating a molecular formula of C_15_H_28_O_9_ (calcd for C_15_H_27_O_9_; 351.1655) and two degrees of unsaturation. The IR spectrum showed absorptions for hydroxyl (3376 cm^−1^) and carbonyl (1725 cm^−1^) functionalities. Fifteen carbon signals, including two methyls, five methylenes, seven methines, and one quaternary carbon, were observed in the one-dimensional (1D) NMR spectra of **1** (Table 1). The quaternary carbon was identified as a carbonyl carbon on the basis of the chemical shift at *δ*_C_ 176.6. The 1D and HSQC (heteronuclear single quantum coherence) NMR spectra displayed one anomeric (*δ*_H_/*δ*_C_: 4.27 (d, *J* = 8.0 Hz)/104.9), five oxymethine (*δ*_H_/*δ*_C_: 3.21 (t, *J* = 8.0 Hz)/75.1, 3.28 (overlap)/71.6, 3.28 (overlap)/78.0, 3.35 (t, *J* = 8.0 Hz)/77.9, and 3.89 (td, *J* = 7.0, 2.5 Hz)/73.6), and three oxymethylene (*δ*_H_/*δ*_C_: 3.57 (dd, *J* = 10.5, 7.0 Hz), 4.01 (dd, *J* = 10.5, 2.5 Hz)/73.1, 3.66 (dd, *J* = 12.0, 5.0 Hz), 3.85 (brd, *J* = 12.0 Hz)/62.7, and 4.10 (2H, t, *J* = 7.5 Hz)/65.5) signals. The HMBC (heteronuclear multiple bond correlation) correlations (Figure 2) of compound **1** at the methine protons H-2 (*δ* 2.67, quint) with C-1 (*δ* 176.6)/C-3 (*δ* 73.6)/C-4 (*δ* 73.1)/C-5 (*δ* 13.9), H-3 (*δ* 3.89, td) with C-1/C-2 (*δ* 44.6)/C-4/C-5, H-1′ (*δ* 4.27, d) with C-4, and the methyl protons H_3_-5 (*δ* 1.14, d) with C-1/C-2/C-3 suggested that the methyl and hydroxyl moieties were positioned at C-2 and C-3, respectively. In addition, the methylene protons at H_2_-1″ (*δ* 4.10, t) and H_2_-4 (*δ* 3.57, dd; 4.01, dd) exhibited ^3^*J* interactions with C-1 and C-1′ (*δ* 104.9), respectively, in the HMBC spectrum (Figure 2) that supported the assignments of the *n*-butyl group at C-1 and one beta-glucose (H-1′, *δ* 4.27, d, *J* = 8.0 Hz) at C-4 [8]. Figure 2 shows key COSY and HMBC correlations observed for **1**. The relative configurations at C-2 and C-3 (3-hydroxy-2-methyl moiety) of **1** could be determined anti-form by the ^3^*J*_H2-H3_ value of 7.0 Hz (anti-form: 7.0 Hz; syn-form: 4.0 Hz) [9]. To determine the absolute configuration, compound **1** was treated separately with (*R*)- and (*S*)-α-methoxy-α-(trifluoromethyl)-phenylacetyl chloride [(*R*)- and (*S*)-MTPA-Cl] in the presence of pyridine-*d5* to obtain the (*S*)- and (*R*)-MTPA esters (**1a** and **1b**), respectively. The MTPA esters were generated successfully at C-3 and C-2′/C-3′/C-4′, as elucidated from the ^1^H NMR and ^1^H-^1^H COSY spectra (**1a**, H-3, *δ* 5.82, m; H-2′, *δ* 5.70, t, *J* = 9.5 Hz; H-3′, *δ* 4.39, t, *J* = 9.5 Hz; H-4′, *δ* 5.76, t, *J* = 9.5 Hz; **1b**, H-3, *δ* 5.81, m; H-2′, *δ* 5.56, t, *J* = 9.5 Hz; H-3′, *δ* 4.12, t, *J* = 9.5 Hz; H-4′, *δ* 5.66, t, *J* = 9.5 Hz). The differences between the ^1^H NMR chemical shifts for **1a** and **1b** (Δ values shown in Figure 3) led to the assignments of *S*- and *R*-configurations at C-3 and C-2, respectively. Consequently, compound **1** was elucidated as butyl 4-β-d-glycopyranosyl-(*S*)-3-hydroxy-(*R*)-2-methylbutanoate and named tuliposide H.

Compound **2**, tuliposide I, gave the molecular formula, C_15_H_28_O_8_, as established by HRESIMS (*m*/*z* 359.1686 [M + Na]^+^, calcd for 359.1682). The 1D NMR spectra of **2** were also similar to those of **1**, except the fact that the chemical shifts of the methylene signal *δ*_H_ 1.65 (sext, *J* = 7.0 Hz) and 1.96 (sext, *J* = 7.0 Hz)/*δ*_C_ 34.6 were identified instead of one methine at C-3 of **1** (Table 1). On the basis of the COSY and HMBC data (Figure 2), compound **2** was postulated to be composed of an isoprenoid, one glucose, and the *n*-butyl groups as well. The HMBC ^3^*J* correlations of H_2_-1″ (*δ* 4.04, 2H, t, *J* = 7.5 Hz/*δ*_C_ 65.4) with C-1 (*δ* 178.5) and H-1′ (*δ* 4.19, d, *J* = 8.0 Hz/*δ*_C_ 104.4) with C-4 (*δ* 68.4) suggested that the *n*-butyl group and one β-glucose moiety [8] were connected at C-1 and C-4, respectively (Figure 2). Key HMBC and COSY correlations are shown in Figure 2. The *R*-configuration of H_3_-5 at C-2 of **2** was determined by acid hydrolysis of **2** to offer corresponding tulipalin compound, 3-methyldihydrofuran-2(3*H*)-one (Figure S28) [8] with a positive optical rotation value ([*α*]^23^_D_ +18.7, CH_2_Cl_2_) (*R*: [*α*]^25^_D_ +14.7, CHCl_3_) [10]. The structure of compound **2** was identified as butyl 4-β-d-glycopyranosyl-(*R*)-2-methylbutanoate.

The molecular formula of **3** was deduced as C_11_H_20_O_8_ due to the appearance of a [M + Na]^+^ ion at *m*/*z* 303.1049 (calcd for 303.1050) in the HRESIMS. Eleven carbon signals, including one methyl (*δ* 17.6), three methylenes (*δ* 34.7, 62.8, and 68.6), six methines (*δ* 37.6, 71.6, 75.1, 77.9, 78.0, and 104.4), and one quaternary carbon (*δ* 180.6), were observed in the 1D NMR spectra of **3** (Table 1). The quaternary carbon was identified as a carbonyl carbon on the basis of the carbon chemical shift at *δ* 180.6. Furthermore, compound **3** showed similar spectroscopic data to that of **2**, except the *n*-butyl signals. In the HMBC spectrum (Figure S15), the anomeric proton at *δ* 4.23 (d, *J* = 8.0 Hz) exhibited ^3^*J* interaction with C-4 (*δ* 68.6) led to the β-glucose located at C-4. Key HMBC and COSY connections are shown in Figure 2. Compound **3** was not isolated in a sufficient quantity to determine the absolute configuration at C-2. Finally, tuliposide J (**3**), 4-β-D-glycopyranosyl-(*R*)-2-methylbutanoic acid, was identified because of abovementioned structural elucidations of **1** and **2** in this study.

Compounds **1**–**3** are tuliposides, a kind of specialized products consisting of 4-hydroxy-2-methylenebutanoic acid (HMBA) and/or (3*S*)-3,4-dihydroxy-2-methylenebutanoic acid (DHMBA) groups acylated at C-1 and/or C-6 positions of a β-d-glucose (Glc) mainly found in the genus *Tulipa* [8,11]. So far, tuliposide analogues, including 1-tuliposide A, 6-tuliposide A, 1-tuliposide B, 6-tuliposide B, and tuliposides D–G, have been isolated from *Tulipa* [8,11]. However, the structure of tuliposide C has not been reported and could be missing in previous literatures [11]. Tulipalins, such as tulipalin A and (–)-tulipalin B, are the aglycone portions of tuliposides, which spontaneously lactonizes after releasing HMBA and/or DHMBA groups by tuliposide-converting enzymes [8,11]. In this study, compounds **1**–**3** named as tuliposides H–J belong to tuliposides, but their double bonds at C-2 were reduced and the β-D-glucose groups were attached to C-4 (oxymethylene), instead of C-1 (*O*-acyl functionality) in HMBA or DHMBA (Figure 1). Additionally, compound **10** (2*S*,3*S*) is tulipalin but could not be metabolized from **1** (2*R*,3*S*) due to the different configuration at C-2. The possible biosyntheses of tuliposides **1**–**3** are shown in Figure S29 [8,11].

In addition, 11 known compounds including three pyroglutamic acid analogues (**4**–**6**), three citric acid derivatives (**7**–**9**), two furanones (**10**–**11**), one furan (**12**), and two steroids (**13**–**14**) were isolated from *A. edulis* for the first time. Compounds **1**–**9** and **10**–**14** were obtained from TEB and TEE crude fractions, respectively. They were identified as (+)-pyroglutamic acid (**4**) [12], (+)-methyl 5-oxopyrrolidine-2-carboxylate (**5**) [12], (+)-butyl 5-oxopyrrolidine-2-carboxylate (**6**) [12], 1-butyl citrate (**7**) [13], 1,1′-dibutyl 5-methyl citrate (**8**) [13], tributyl citrate (**9**) [13], (–)-3-hydroxy-2-methylbutyrolactone (**10**) [14,15,16], (–)-methyl 3-hydroxy-5-oxotetrahydrofuran-3-carboxylate (**11**) [17], 5-(hydroxymethyl)furfural (**12**) [18], sitosterol (**13**) [19], and sitosterol-3-β-D-glucose (**14**) [20] from the spectroscopic data and compared with the literature.

### 2.2. Chemical Components Elucidation of TEW

The ^1^H and ^13^C NMR spectra of TEW, a water-soluble crude fraction from TEM extract, showed very similar to that of carbohydrates (Figures S16 and S17). Polysaccharide prepared from this species have been reported to possess rhamnose, xylose, arabinose, galactose, mannose, glucose, and fructose after monosaccharide analysis [3,8]. The NMR spectroscopic data and the TLC (thin-layer chromatography) retention factor of TEW compared with those of the sugar standards (Figures S18–S23) suggested D-glucose and D-fructose were the main components in TEW extracts.

### 2.3. Effects of Extracts and Isolates on Melanogenesis

**Figure 4:**
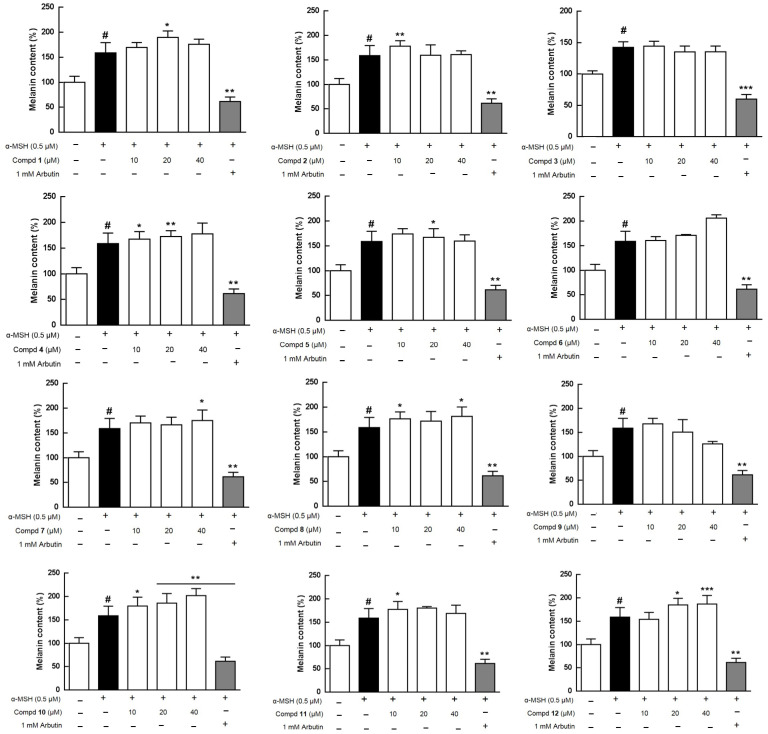
Regulation of melanogenesis effects of compounds **1**–**12**. The data are representative of three independent experiments. #, *p* < 0.05 compared with the control group; *, *p* < 0.05, **, *p* < 0.01, and ***, *p* < 0.001 compared with the α-MSH-treated group.

**Figure 5:**
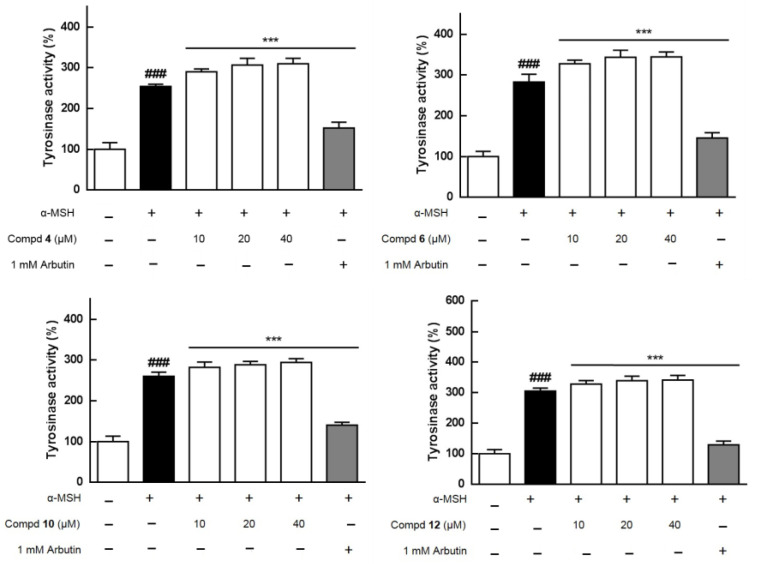
Tyrosinase activity effects of compounds **4**-, **6**-, **10**-, and **12**-treated B16 melanoma cells. ###, *p* < 0.05 compared with the control group; ***, *p* < 0.001 compared with the α-MSH-treated group.

The MeOH extract (TEM) of this species was separated into TEE, TEB, and TEW crude fractions, and the aforementioned four extracts were evaluated for cytotoxicity and melanogenesis effects in B16 melanoma cells at concentrations from 12.5 to 200 μg/mL (Figure S24). TEE showed obvious cytotoxicity toward B16 at 200 μg/mL and TEB as well as TEW had cytotoxic activities at concentrations from 12.5 to 200 μg/mL (Figure S24A-1,B-1,C-1,D-1). In the bioassay, α-melanocyte-stimulating hormone (α-MSH) was used to activate B16 cells to synthesize more melanin, and TEM could moderately stimulate melanogenesis (Figure S24A-2). Most of all isolates (Figure 1), excluding **13** and **14**, were further assayed for the regulation of melanogenesis effects at concentrations from 10 to 40 μM, with arbutin used as a positive control (Figure 4). As shown in Figure 4, compound **9** obviously and dose-dependently inhibited melanin production that resulted from cytotoxicity toward B16 cells with an IC_50_ (half maximal inhibitory concentration) value of 81.9 μM (Figure S25). Isolates **4**, **6**, **10**, and **12** increased melanin contents dose-dependently up to the maximum percentages of 11.9%, 29.8%, 27.2%, and 17.6%, respectively, at 40 μM with no cellular toxicity. Tyrosinase plays a key role in the first two steps of melanogenesis [6]; therefore, the effects of compounds **4**, **6**, **10**, and **12** on intracellular tyrosinase were further evaluated. α-MSH increased the tyrosinase activity up to 254.3–305.5% when compared with the control group (100%) (Figure 5 and Table S1). Consequently, **4** (14.1–21.9%), **6** (15.8–21.9%), **10** (8.5–13.1%), and **12** (7.5–11.8%) moderately increased the activity of the enzyme, tyrosinase, in a dose-dependent manner at concentrations of 10 to 40 μM, while compared with the α-MSH group (Figure 5 and Table S1). A correlation graph between the cellular melanin contents and the effects on the tyrosinase of compounds **4**, **6**, **10**, and **12** is shown in Figure S26. Furthermore, to confirm the melanogenesis-inducing effects of **4**, **6**, **10**, and **12**, each compound was added in B16 cells without co-treated α-MSH that was used as a positive control in this assay (Figure S27). Consequently, individual **4**, **6**, **10**, and **12** did not increase melanin productions alone (Figure S27) similar to those shown in Figure 4, and it was suggested the abovementioned four compounds could merely enhance melanogenesis effects of α-MSH. Accordingly, additional signal pathways in melanogenesis, such as tyrosinase-related protein-1 (TRP-1), TRP-2, microphthalmia-associated transcription factor, melanocortin 1 receptor, cyclic adenosine monophosphate, protein kinase A, cAMP-response element-binding protein, c-Jun N-terminal kinases, extracellular signal-regulated kinase, p38, and phosphoinositide 3-kinase/protein kinase B [7,21], should be further tested and confirmed for those active components.

## 3. Materials and Methods

### 3.1. General Experimental Procedures

NMR spectra were measured on a Bruker Avance III 500 MHz instrument (Bruker, Billerica, America) for ^1^H and 125 MHz for ^13^C-NMR. Chemical shift (*δ*) values were in ppm, and coupling constants (*J*) were in Hz with CD_3_OD, CDCl_3_, and/or D_2_O used as solvents. Optical rotations were obtained on a JASCO-P-2000 polarimeter (JASCO, Tokyo, Japan) (cell length 10 mm). IR spectra were recorded on a PerkinElmer Spectrum Two FT-IR spectrometer (PerkinElmer, Waltham, MA, USA). Low- and high-resolution ESIMS (electrospray ionization mass spectrometry) were measured on a Bruker Daltonics Esquire HCT ultra high capacity trap mass spectrometer and an Orbitrap mass spectrometer (LTQ Orbitrap XL, Thermo Fisher Scientific), respectively. TLC was performed on Kieselgel 60 F_254_ (0.25 mm; Merck) and/or RP-18 F_254_S (0.25 mm; Merck), coated plates and then stained by spraying with 5% (*v*/*v*) sulfuric acid in a MeOH solution and heating on a hot plate. Silica gel (Silicycle: 70–230 and 230–400 mesh), RP-18 (LiChroprep^®^ 40–63 µm; Merck), Sephadex^TM^ LH-20 (GE Healthcare, Uppsala, Sweden), Diaion^®^ HP-20 (Supelco^TM^, Bellefonte), and MCI^®^ CHP20P (Supelco^TM^, Bellefonte) were applied for column chromatography. A Shimadzu LC-20AT pump and a Shimadzu RID-10A refractive index detector (Shimadzu Inc., Kyoto, Japan), along with a Cosmosil 5C_18_-MS-II (250 × 10 mm i.d., 5 µm) column at a flow rate of 2.0 mL/min, were used for HPLC. Sugar reagents, including D-glucose (MP Biomedicals, LLC, Illkirch, France) and D-fructose (TCI, Tokyo, Japan) were used for water-soluble crude fraction (TEW) analysis.

### 3.2. Plant Material

Dry bulbs of *A. edulis* (formerly *T. edulis*) were purchased from a traditional Chinese medicine pharmacy in Taichung, Taiwan, in September 2018 and identified by the author, Prof. Chang. A voucher specimen (TE201809) was deposited at the Chinese Medicine Research and Development Center, CMUH, Taiwan.

### 3.3. Extraction and Isolation

The bulbs of *A. edulis* (5.0 kg) were extracted eight times with MeOH (8.0 L each) at room temperature to obtain a crude extract. The MeOH extract (TEM, 525.0 g) was partitioned three times between ethyl acetate (EtOAc) and H_2_O (1500:1500, *v*/*v*) to give an EtOAc-soluble fraction (TEE, 45.0 g) and an aqueous phase, which was further partitioned with *n*-BuOH/H_2_O (1500:1500, *v*/*v* × 3), and then separated into *n*-BuOH-soluble (TEB, 53.6 g) and H_2_O-soluble (TEW, 410.0 g) fractions.

TEE was subjected to open column chromatography on a silica gel (0.063–0.200 mm, column: 7 × 26 cm, diameter × length), using gradients of hexane–EtOAc–MeOH (30:1:0; 1:1:0; 0:0:1, *v*/*v*/*v*) and gave 14 subfractions (TEE1–TEE14). Precipitate **14** (249.0 mg; isolation yield: 0.00498%) obtained from subfraction TEE12 (2.2 g) was filtered and washed with MeOH. TEE9 (3.6 g) was fractionated into seven subfractions (TEE9-1 to 9-7) by silica gel (column: 5 × 24 cm; CH_2_Cl_2_–MeOH, 7:1; 1:1; 0:1, *v*/*v*), with subfraction TEE9-4 (1.1 g), and then subjected to Sephadex LH-20 column chromatography (column: 5 × 54 cm; CH_2_Cl_2_–MeOH, 1:1 to 0:1, *v*/*v*), to give seven subfractions (TEE9-4-1 to 9-4-7). TEE9-4-5 and TEE9-4-6 were combined (total 551.7 mg) and repeatedly purified by RP-HPLC (MeOH–H_2_O, 45:55; 20:80, *v*/*v*) to give **12** (21.0 mg; *t*_R_ = 13 min; isolation yield: 0.00042%) and a mother liquor (131.2 mg), which was further subjected to RP-HPLC (MeOH–H_2_O; 10:90, *v*/*v*) to give compounds **10** (35.5 mg; *t*_R_ = 13 min; isolation yield: 0.00071%) and **11** (1.2 mg; *t*_R_ = 15 min; isolation yield: 0.000024%). Fraction TEE6 (2.7 g) was isolated by Sephadex LH-20 (column: 2.5 × 45 cm; CH_2_Cl_2_–MeOH, 1:1; 0:1, *v*/*v*) to get **13** (6.8 mg; isolation yield: 0.000136%).

TEB was chromatographed over a Diaion HP-20 column (6 × 60 cm; H_2_O–MeOH–acetone, 100:0:0; 25:75:0; 50:50:0; 75:25:0; 0:100:0; 0:0:100, *v*/*v*/*v*) to give six subfractions (TEB1–TEB6). Subfraction TEB5 (502.0 mg) was purified by RP-HPLC (MeOH–H_2_O, 85:15, *v*/*v*) to obtain compounds **8** (9.3 mg; *t*_R_ = 10 min; isolation yield: 0.000186%) and **9** (144.2 mg; *t*_R_ = 15 min; isolation yield: 0.002884%). Fraction TEB3 (5.5 g) was subjected to RP-18 chromatography (column: 7 × 25 cm; MeOH–H_2_O, 1:1; 1:0, *v*/*v*), and subfraction TEB3-6 (1.0 g) was further isolated by Sephadex LH-20 (column: 5 × 54 cm; MeOH) to obtain seven subfractions (TEB3-6-1 to 3-6-7). TEB3-6-4 (377.1 mg) was separated by MCI gel chromatography (column: 2.5 × 23 cm; H_2_O–MeOH, 100:0; 80:20; 60:40; 40:60; 20:80; 0:100, *v*/*v*) to give seven subfractions (TEB3-6-4-1 to 3-6-4-7). TEB3-6-4-4 (38.1 mg) was purified by RP-HPLC (MeOH–H_2_O, 45:55 in 0.1% formic acid, *v*/*v*) to afford pure **1** (12.2 mg; *t*_R_ = 17 min; isolation yield: 0.000244%). Additionally, TEB3-6-4-6 (31.0 mg) was conducted with RP-HPLC (MeOH–H_2_O, 40:60 in 0.1% formic acid, *v*/*v*) to yield **6** (7.1 mg; *t*_R_ = 28 min; isolation yield: 0.000142%). TEB3-6-5 (410.7 mg) was isolated by MCI gel chromatography (column: 2.5 × 23 cm; H_2_O–MeOH, 100:0; 80:20; 60:40; 50:50; 40:60; 30:70; 20:80; 0:100, *v*/*v*) to give eight subfractions (TEB3-6-5-1 to 3-6-5-8). TEB3-6-5-3 (75.1 mg) was purified by MCI gel chromatography (column: 1 × 20 cm; H_2_O–MeOH, 80:20; 70:30; 0:100, *v*/*v*) to afford compounds **4** (59.3 mg; isolation yield: 0.001186%) and **5** (10.2 mg; isolation yield: 0.000204%). Subfraction TEB3-6-5-4 (97.0 mg) was purified by RP-HPLC (MeOH–H_2_O, 40:60 in 0.1% formic acid, *v*/*v*) to obtain **7** (46.4 mg; *t*_R_ = 18 min; isolation yield: 0.000928%). TEB3-10 (925.4 mg) was chromatographed over an MCI gel chromatography (column: 2.5 × 28 cm; H_2_O–MeOH, 100:0; 80:20; 60:40; 50:50; 40:60; 30:70; 10:90; 0:100, *v*/*v*) to give eight subfractions (TEB3-10-1 to 3-10-8) and pure **2** (393.4 mg; isolation yield: 0.007868%). Compound **3** (6.3 mg; *t*_R_ = 8 min; isolation yield: 0.000126%) was obtained from TEB3-10-2 (20.9 mg) by RP-HPLC purification (MeOH–H_2_O, 30:70 in 0.1% formic acid, *v*/*v*).

#### 3.3.1. Tuliposide H (**1**)

[α]^23^_D_ −23.1 (c 0.12, MeOH); IR (neat) ν_max_ 3376 (OH), 2960, 2934, 2875 (CH), 1725 (C=O), 1640, 1589, 1459, 1382, 1259, 1167, 1075, 1041 (C–O–C), 926, 900, 632, 521 cm^−1^; ^1^H and ^13^C NMR spectroscopic data, see Table 1; HRESIMS *m*/*z* 351.1652 [M − H]^−^ (calcd for C_15_H_27_O_9_, 351.1655).

#### 3.3.2. Tuliposide I (**2**)

[*α*]^23^_D_ −25.8 (*c* 0.2, MeOH); IR (neat) *ν*_max_ 3404 (OH), 2961, 2934, 2876 (CH), 1729 (C=O), 1638, 1461, 1378, 1277, 1185, 1165, 1077, 1036 (C–O–C), 897, 736, 625, 517 cm^−1^; ^1^H and ^13^C NMR spectroscopic data, see Table 1; HRESIMS *m*/*z* 359.1686 [M + Na]^+^ (calcd for C_15_H_28_O_8_Na, 359.1682).

#### 3.3.3. Tuliposide J (**3**)

[*α*]^23^_D_ +9.0 (*c* 0.1, MeOH); IR (neat) *ν*_max_ 3424 (OH), 2953, 2924, 2853 (CH), 1740 (C=O), 1632, 1441, 1401 (C–O–C), 1284, 1205, 1182, 1120, 1078, 1039, 893, 768, 721 cm^−1^; ^1^H and ^13^C NMR spectroscopic data, see Table 1; HRESIMS *m*/*z* 303.1049 [M + Na]^+^ (calcd for C_11_H_20_O_8_Na, 303.1050).

### 3.4. (R)- and (S)-MTPA Derivatives of **1**

The preparation of the (*S*)-MTPA ester derivative of **1** was carried out by a convenient Mosher ester procedure [22,23]. Compound **1** (3.4 mg, 0.01 mmole) was transferred into a clean NMR tube and then dried completely under vacuum before being full of nitrogen. Furthermore, C_5_D_5_N (0.5 mL) and (*R*)-(−)-α-methoxy-α-(trifluoromethyl)-phenyl-acetyl chloride (MTPA chloride, 12.2 mg, 0.05 mmole) were added immediately into the sealed NMR tube that was further shaken carefully to mix the sample and MTPA chloride. The ^1^H NMR and ^1^H-^1^H COSY spectra of the mixture after reacting for 24 h at room temperature were recorded. The (*R*)-MTPA ester of **1** was also prepared similarly by the aforementioned process. Compound **1**: 1H NMR (C_5_D_5_N, 500 MHz) *δ* 0.78 (H-4′′, t), 1.26 (H-3′′, sext), 1.28 (H3-5, d), 1.52 (H-2′′, quint), 3.08 (H-2, quint), 3.95 (H-5′, m), 4.03 (H-4a, dd), 4.05 (H-2′, overlap), 4.15 (H-1′′, t), 4.23 (H-3′, overlap), 4.23 (H-4′, overlap), 4.36 (H-6′a, dd), 4.39 (H-3, overlap), 4.43 (H-4b, dd), 4.53 (H-6′b, d), and 4.95 (H-1′, d).

#### 3.4.1. (*S*)-MTPA Ester of **1** (**1a**)

^1^H NMR (C_5_D_5_N, 500 MHz) *δ* 0.77 (H-4′′, t), 1.17 (H-3′′, sext), 1.21 (H3-5, d), 1.37 (H-2′′, quint), 3.18 (H-2, quint), 3.92 (H-1′′, t), 3.97 (H-4a, dd), 4.33 (H-5′, m), 4.39 (H-3′, t), 4.49 (H-4b, brd), 4.64 (H-6′a, dd), 4.96 (H-1′, d), 5.05 (H-6′b, d), 5.70 (H-2′, t), 5.76 (H-4′, t), and 5.82 (H-3, m).

#### 3.4.2. (*R*)-MTPA Ester of **1** (**1b**)

^1^H NMR (C_5_D_5_N, 500 MHz) *δ* 0.79 (H-4′′, t), 1.26 (H-3′′, sext), 1.30 (H3-5, d), 1.50 (H-2′′, quint), 3.33 (H-2, quint), 3.60 (H-5′, m), 3.94 (H-4a, dd), 4.11 (H-1′′, t), 4.12 (H-3′, t), 4.13 (H-6′a, dd), 4.50 (H-4b, brd), 4.70 (H-1′, d), 4.85 (H-6′b, d), 5.56 (H-2′, t), 5.66 (H-4′, t), and 5.81 (H-3, m).

### 3.5. Acid Hydrolysis of Tuliposide I (**2**)

Isolate **2** (78.6 mg) was hydrolyzed in 1 M HCl (1.5 mL) at 100 °C for 2 h [8]. After cooling, the reaction mixture was extracted with CH_2_Cl_2_ (2.0 mL × 3) to offer tulipalin analogue, 3-methyldihydrofuran-2(3*H*)-one (11.1 mg). [*α*]^23^_D_ +18.7 (*c* 1.1, CH_2_Cl_2_); ^1^H NMR (CDCl_3_, 400 MHz) *δ* 1.30 (3H, d, *J* = 7.0 Hz), 1.94 (m), 2.45 (m), 2.61 (m), 4.20 (ddd, *J* = 8.8, 6.8, 6.4 Hz), 4.33 (ddd, *J* = 8.8, 8.8, 2.8 Hz). ^13^C NMR (CDCl_3_, 100 MHz) *δ* 15.2, 30.7, 34.1, 66.2, 180.1 (Figure S28); ESIMS *m*/*z* 101.06 [M + H]^+^.

### 3.6. Cell Culture

The murine B16 melanoma cells were cultured in Dulbecco’s Modified Eagle Medium (DMEM; GIBCO Invitrogen corporation, New York, NY, USA) supplemented with 10% fetal bovine serum, 100 U/mL of penicillin, and 100 μg/mL of streptomycin at 37 °C in a 5% CO_2_ incubator.

### 3.7. Measurement of B16 Cell Viability

The cytotoxicity of B16 cells for the test compounds was measured by MTT with a previously described protocol with slight modifications [6]. The cells were seeded in 96-well plates (1 × 10^3^ cells/well) and cultured for 24 h before treated with compounds for other 72 h. After that, the medium was removed, 100 μL of MTT reagent (Thermo Scientific, Waltham, MA, USA) (at the final concentration of 0.5 mg/mL) was added to each well and then incubated at 37 °C for 1 h. The formazan crystal was dissolved in 100 μL DMSO, and the optical density was measured by using a microplate reader (SPECTORstar^®^ Nano, BMG LABTECH, Ortenberg, Germany) at 570 nm.

### 3.8. Measurement of Melanin Content in B16 Cells

The melanin content in B16 cells was assayed according to a previously described method with slight modifications [6]. The cells were seeded at a density of 5 × 10^3^ cells/well in 24-well plates for 24 h. The medium was replaced by a 500 μL fresh culture medium containing 0.5 μM α-MSH with or without compounds for 72 h. Arbutin (1 mM) was used as the positive control. After that, the medium was removed and washed by PBS (pH 6.8) twice. The cells were harvested by NaOH (200 μL, 2 N) and then heated at 85 °C for 30 min. After cooling to room temperature and being centrifuged, the absorbance was measured at 405 nm using a microplate reader.

### 3.9. Intracellular Tyrosinase Activity

Intracellular tyrosinase activity assay was performed by a slightly modified method, which was reported previously [6]. The cells were seeded in 24-well plates at a density of 5 × 10^3^ cells/well for 24 h. The cells were treated in DMEM containing 0.5 μM α-MSH with or without compounds for 72 h. After removing the medium, the cells were washed by cold PBS (pH 6.8) twice. The cells were lysed by 100 μL lysis buffer (1% triton X-100 in PBS) and frozen at −80 °C for 15 min. Then, the cell lysates were centrifuged at 13,200× *g* at 4 °C for 30 min to obtain the supernatant. The total protein content of the supernatant was quantified by BCA protein assay. Next, each quantified lysate (30 μg/90 μL) was mixed with 10 μL of L-DOPA (15 mM) in a 96-well plate and then incubated at 37 °C for 1 h, and the absorbance was measured at 405 nm using a microplate reader.

## 4. Conclusions

Overall, 14 pure compounds, including three new isoprenoid glycosides, tuliposides H–J (**1**–**3**), were isolated from *A. edulis*. Citric acid derivative **9**, tributyl citrate, had significantly anti-melanogenesis activity but showed cytotoxicity toward B16 melanoma cell that could be further researched for anti-melanoma drugs. While pyroglutamic acid analogues **4** and **6**, furanone **10**, and furan **12** had dose-dependently increased melanin contents and activated tyrosinase that could be further studied for vitiligo skin disease or anti-whitening and anti-hypopigmentation agents for hair. However, in vitro mechanisms and/or in vivo studies are needed to be investigated in more detail to verify the efficacy of the active components.

## Figures and Tables

**Table 1:** ^1^H and ^13^C NMR spectroscopic data (500 and 125 MHz; CD_3_OD) for **1**–**3**.

	Tuliposide H (1)		Tuliposide I (2)		Tuliposide J (3)	
Position	*δ*_H_ (*J* in Hz)	*δ*_C_	*δ*_H_ (*J* in Hz)	*δ*_C_	*δ*_H_ (*J* in Hz)	*δ*_C_
Isoprenoid						
1		176.6		178.5		180.6
2	2.67 (quint, 7.0)	44.6	2.63 (sext, 7.0)	37.8	2.63 (sext, 7.0)	37.6
3	3.89 (td, 7.0, 2.5)	73.6	1.65 (sext, 7.0) 1.96 (sext, 7.0)	34.6	1.68 (sext, 7.0) 2.00 (sext, 7.0)	34.7
4	3.57 (dd, 10.5, 7.0) 4.01 (dd, 10.5, 2.5)	73.1	3.54 (dt, 10.0, 7.0) 3.87 (dt, 10.0, 7.0)	68.4	3.61 (dt, 10.0, 7.0) 3.92 (dt, 10.0, 7.0)	68.6
5	1.14 (d, 7.0)	13.9	1.12 (d, 7.0)	17.5	1.17 (d, 7.0)	17.6
Glc						
1′	4.27 (d, 8.0)	104.9	4.19 (d, 8.0)	104.4	4.23 (d, 8.0)	104.4
2′	3.21 (t, 8.0)	75.1	3.14 (t, 8.0)	75.6	3.16 (t, 8.0)	75.1
3′	3.35 (t, 8.0)	77.9	3.30 (t, 8.0)	78.0	3.34 (t, 8.0)	77.9
4′	3.28 (overlap)	71.6	3.22 (overlap)	71.4	3.27 (overlap)	71.6
5′	3.28 (overlap)	78.0	3.22 (overlap)	77.8	3.27 (overlap)	78.0
6′	3.66 (dd, 12.0, 5.0) 3.85 (brd, 12.0)	62.7	3.62 (dd, 12.0, 5.5) 3.80 (dd, 12.0, 2.0)	62.8	3.66 (dd, 12.0, 5.5) 3.85 (brd, 12.0)	62.8
*n*-butyl						
1″	4.10 (2H, t, 7.5)	65.5	4.04 (2H, t, 7.5)	65.4		
2″	1.63 (2H, quint, 7.5)	31.8	1.57 (2H, quint, 7.5)	31.8		
3″	1.41 (2H, sext, 7.5)	20.2	1.34 (2H, sext, 7.5)	20.2		
4″	0.94 (3H, t, 7.5)	14.0	0.90 (3H, t, 7.5)	14.0		

## Data Availability

Not applicable.

## Supplementary Materials

The following are available online, Figures S1–S5: NMR spectral data of compound **1**, Figures S6–S10: NMR spectral data of compound **2**, Figures S11–S15: NMR spectral data of compound **3**, Figures S16–S22: NMR spectral data of TEW, Figure S23: TLC analysis of TEW, Figure S24: Cytotoxicity and the regulation of melanogenesis effects of TEM, TEE, TEB, and TEW, Figure S25: Cytotoxicity data of compounds **1**–**12**, Figure S26: A correlation graph between the cellular melanin contents and the effects on tyrosinase of compounds **4**, **6**, **10**, and **12**, Figure S27: Melanogenesis effects of compounds **4**, **6**, **10**, and **12** alone without α-MSH, Figure S28: NMR spectral and optical rotation data of the product obtained from acid hydrolysis of compound **2**, Figure S29: Possible biosynthesis of compounds **1**−**3**, Table S1: Tyrosinase activity effects of compounds **4**, **6**, **10**, and **12**.

## Sample Availability

Samples of the compounds **1**–**14** are available from the authors.
