# Supplementary material for: Tuliposides H–J and Bioactive Components from the Bulb of Amana edulis
Source: Molecules. 2021 Sep 29;26(19):5907. doi: 10.3390/molecules26195907 (PMC8512022; doi:10.3390/molecules26195907)
Supplement: Supplementary file 1 [file molecules-26-05907-s001.zip › molecules-1404810-supplementary.pdf]

Supplementary Materials for

Tuliposides H–J and Bioactive Components from the Bulb of *Amana edulis*

Chia-Lin Lee [1,2,3,*], Zhi-An Gao [1], Yun-Lian Jhan [1], Yuan-Shiun Chang [4] and Chao-Jung Chen [5,6]

[1] Department of Cosmeceutics, China Medical University, Taichung 406040, Taiwan; chlilee@mail.cmu.edu.tw (C.L.L.); s10061131@gmail.com (Z.A.G.); u9884002@cmu.edu.tw (Y.L.J.)

[2] Chinese Medicine Research and Development Center, China Medical University Hospital, Taichung 40402, Taiwan

[3] Chinese Medicine Research Center, China Medical University, Taichung 40402, Taiwan

[4] Department of Chinese Pharmaceutical Sciences and Chinese Medicine Resources, China Medical University, Taichung 40402, Taiwan; yschang@mail.cmu.edu.tw

[5] *Graduate Institute of Integrated Medicine,* China Medical University, Taichung 40402, Taiwan; ironmanchen@yahoo.com.tw

[6] Proteomics Core Laboratory, Department of Medical Research, China Medical University Hospital, Taichung 40402, Taiwan

* Correspondence: chlilee@mail.cmu.edu.tw; Tel.: + 886 4 22053366 ext 5806

Figure S1. ^{1}H NMR (500 MHz, CD_3OD) of compound **1**……………………………..……….....S3
Figure S2. ^{13}C NMR (125 MHz, CD_3OD) of compound **1**……………………………………S3
Figure S3. COSY spectrum (500 MHz, CD_3OD) of compound **1**…………………..…..…………..S4
Figure S4. HSQC spectrum (500 MHz, CD_3OD) of compound **1**…………………………….……..S4
Figure S5. HMBC spectrum (500 MHz, CD_3OD) of compound **1**……………………………………S5
Figure S6. ^{1}H NMR (500 MHz, CD_3OD) of compound **2**…………………………………………......S5
Figure S7. ^{13}C NMR (125 MHz, CD_3OD) of compound **2**……………………………………….....S6
Figure S8. COSY spectrum (500 MHz, CD_3OD) of compound **2**……………………….……………......S6
Figure S9. HSQC spectrum (500 MHz, CD_3OD) of compound **2**…………………………………....S7
Figure S10. HMBC spectrum (500 MHz, CD_3OD) of compound **2**……...…………………………....S7
Figure S11. ^{1}H NMR (500 MHz, CD_3OD) of compound **3**…………….……………………….……S8
Figure S12. ^{13}C NMR (125 MHz, CD_3OD) of compound **3**……….…………..…………..………S8
Figure S13. COSY spectrum (500 MHz, CD_3OD) of compound **3**…………….………………….…S9
Figure S14. HSQC spectrum (500 MHz, CD_3OD) of compound **3**……….…………………………..…S9
Figure S15. HMBC spectrum (500 MHz, CD_3OD) of compound **3**……………………………….…S10
Figure S16. ^{1}H NMR (500 MHz, D_2O with 1 drop CD_3OD) of TEW crude fraction....…………….…S10
Figure S17. ^{13}C NMR (125 MHz, D_2O with 1 drop CD_3OD) of TEW crude fraction....……..…..…S11
Figure S18. COSY spectrum (500 MHz, D_2O with 1 drop CD_3OD) of TEW crude fraction....……..…S11
Figure S19. HSQC spectrum (500 MHz, D_2O with 1 drop CD_3OD) of TEW crude fraction.....….…S12
Figure S20. HMBC spectrum (500 MHz, D_2O with 1 drop CD_3OD) of TEW crude fraction....…..…S12
Figure S21. ^{1}H NMR (500 MHz, D_2O with 1 drop CD_3OD) of D-glucose standard, TEW, and D-fructose standard, respectively....……………...……………………………..……S13
Figure S22. ^{13}C NMR (125 MHz, D_2O with 1 drop CD_3OD) of D-glucose standard, TEW, and D-fructose standard, respectively....………………...……………………….…….S13
Figure S23. TLC analysis of TEW....………………………………………………...…………….……S14
Figure S24. Cytotoxicity toward B16 and the regulation of melanogenesis effects in B16 cells of crude extract TEM, fractions TEE, TEB, and TEW, respectively ……………………..S14
Figure S25. Cytotoxicity data of compounds **1**–**12** toward B16 cells....…………………………..……S15
Figure S26. A correlation graph between the cellular melanin contents and the effects on tyrosinase of compounds **4**, **6**, **10**, and **12**………..………………………..…………S16
Figure S27. Effects of compounds **4**, **6**, **10**, and **12** without co-treated α-MSH on melanin content of B16 melanoma cells…………………………………………………S16
Figure S28. ^{1}H NMR (400 MHz, $CDCl_3$) (A), ^{13}C NMR (100 MHz, $CDCl_3$) (B), and optical rotation data (C) of the product, 3-methyldihydrofuran-2(3*H*)-one obtained from acid hydrolysis of compound **2**………………………………………S17
Figure S29. The possible biosynthesis of compounds **1**−**3**………………………………………...…S18
Table S1. Tyrosinase activity effects of compounds **4**, **6**, **10**, and **12**…………………………………S15

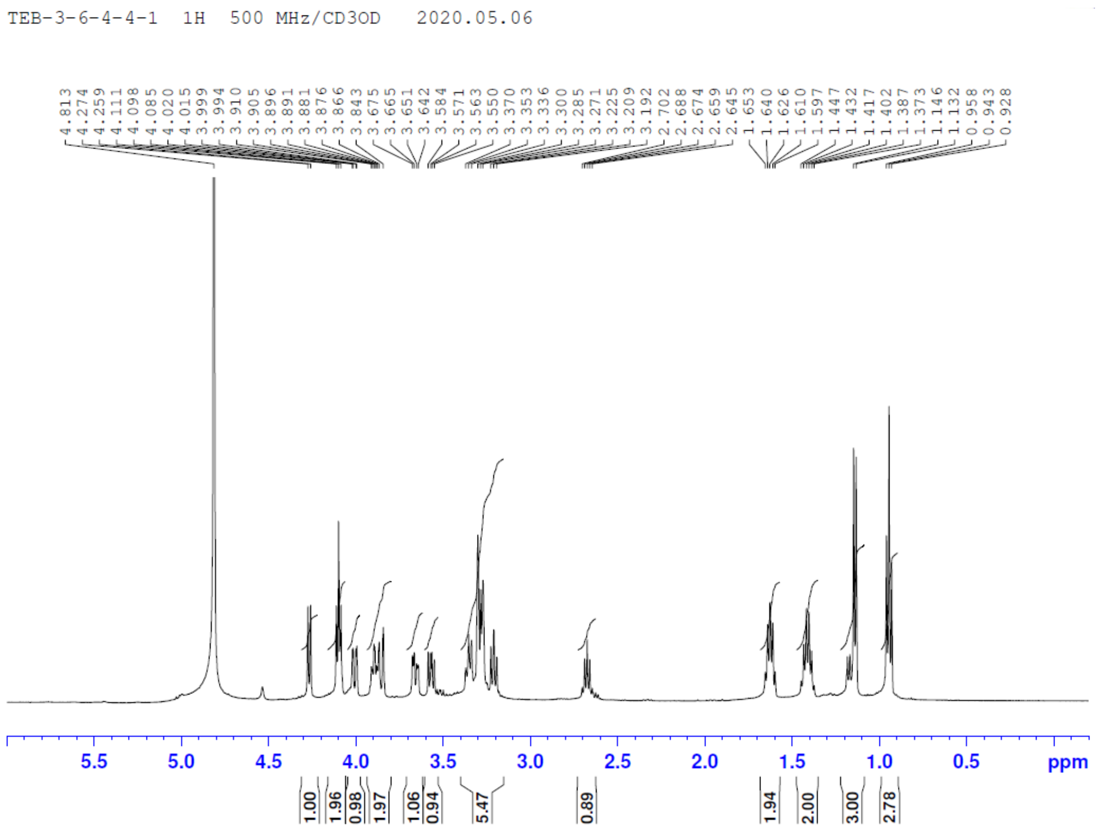

Figure S1. ^{1}H NMR (500 MHz, CD_3OD) of compound **1**.

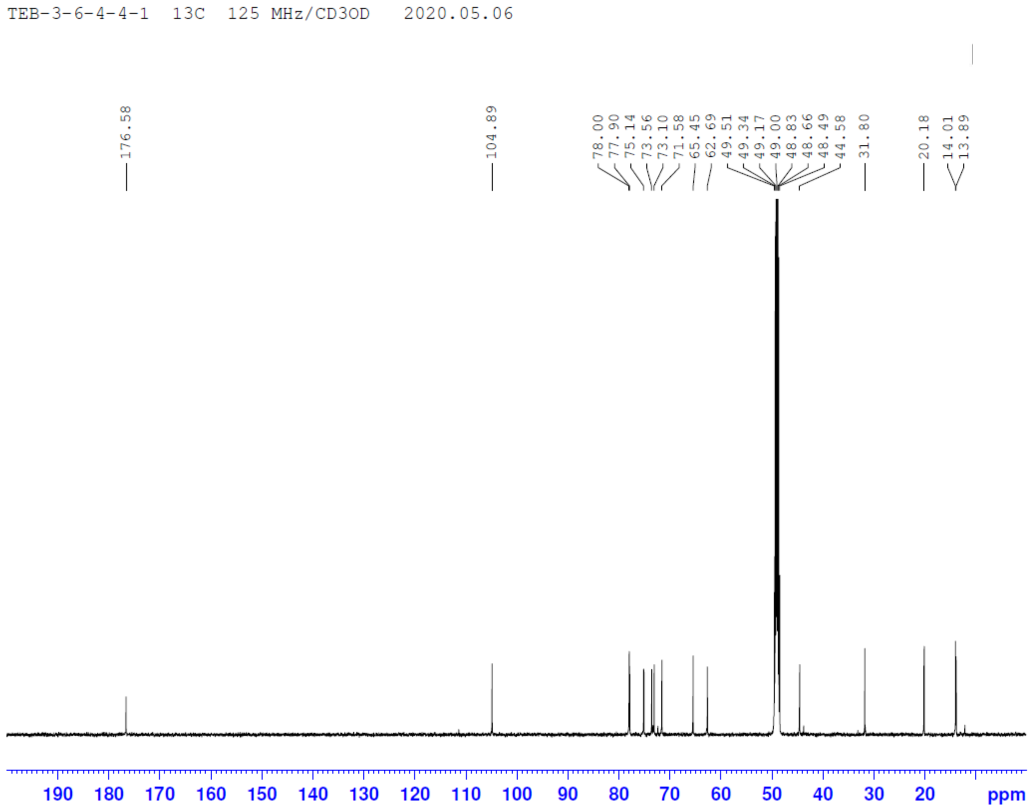

Figure S2. ^{13}C NMR (125 MHz, CD_3OD) of compound **1**.

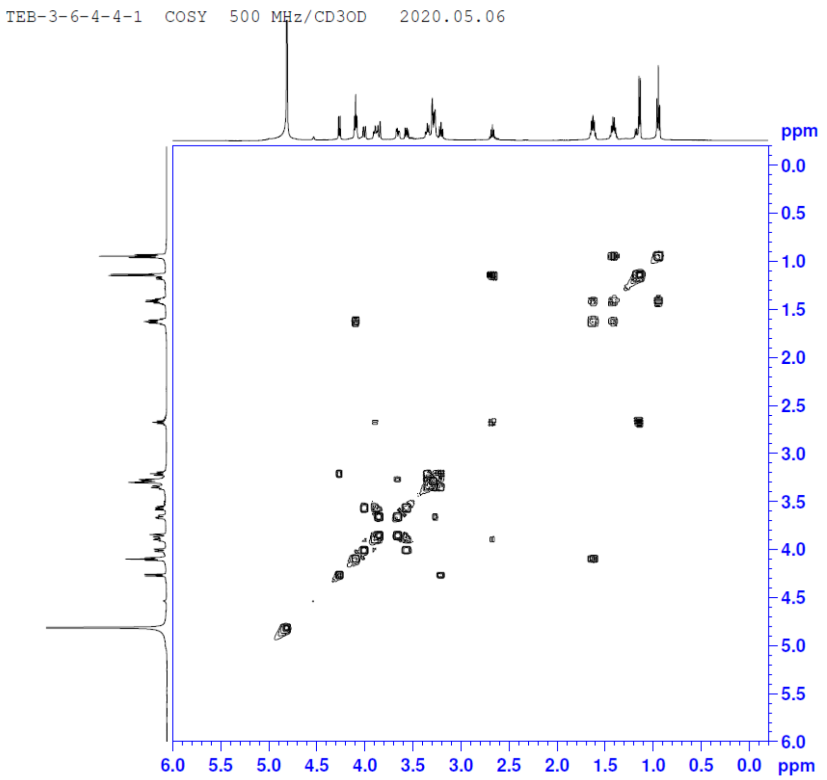

Figure S3. COSY spectrum (500 MHz, CD_3OD) of compound **1**.

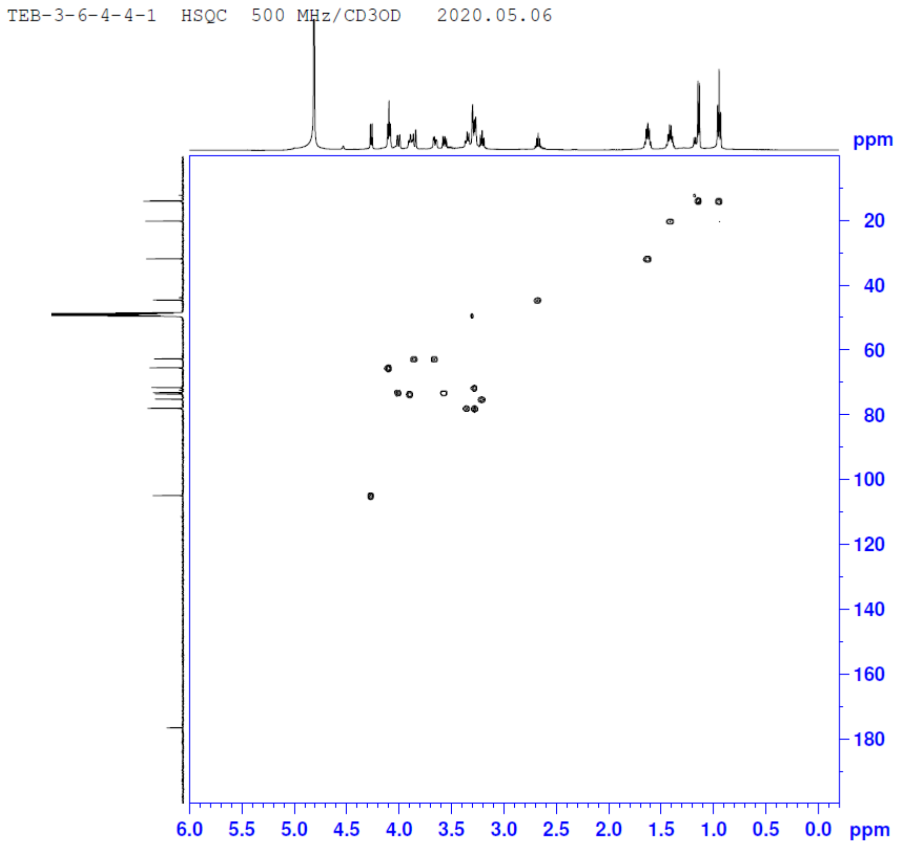

Figure S4. HSQC spectrum (500 MHz, CD_3OD) of compound **1**.

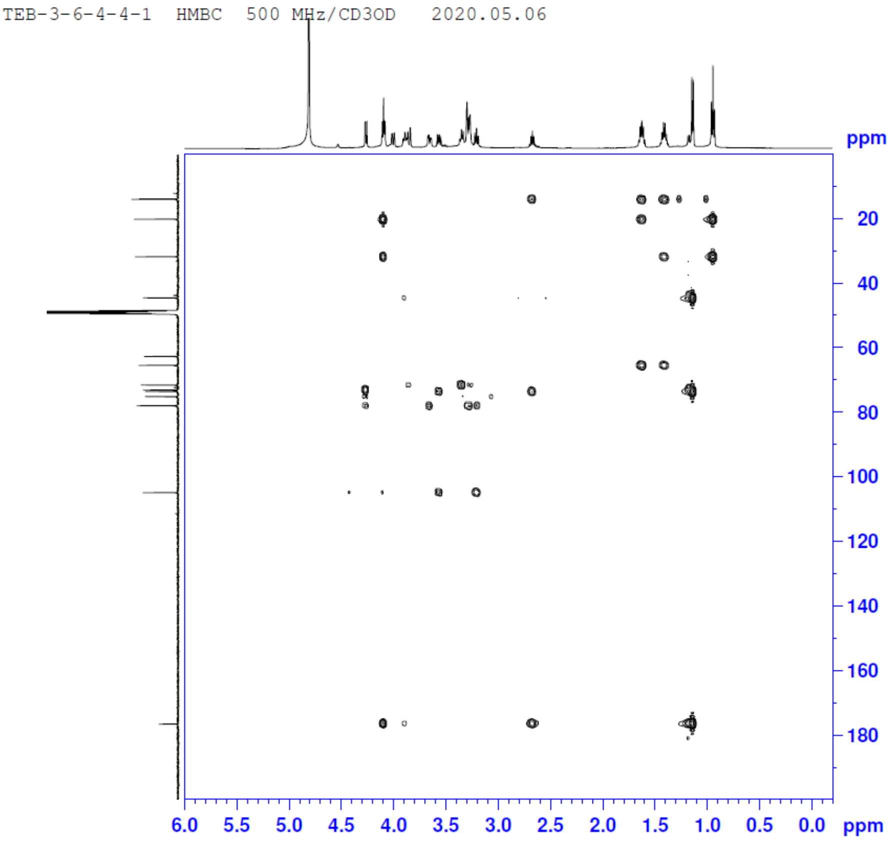

Figure S5. HMBC spectrum (500 MHz, CD_3OD) of compound **1**.

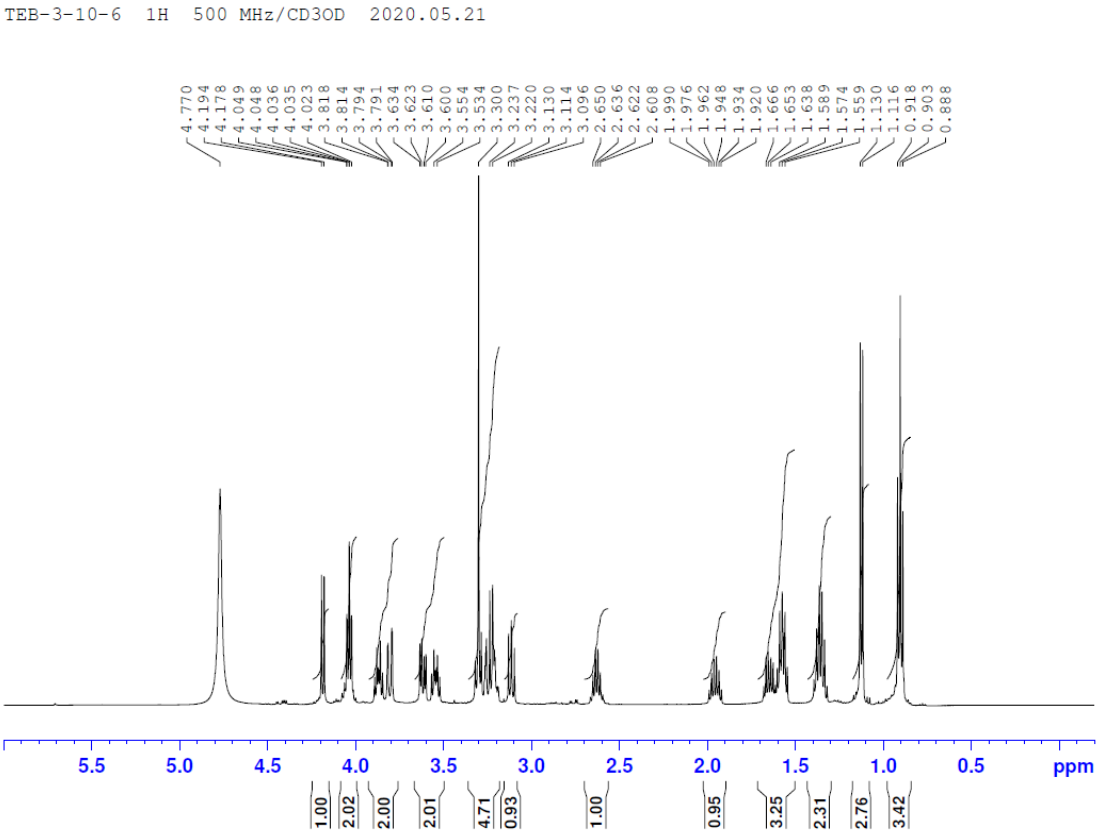

Figure S6. ^{1}H NMR (500 MHz, CD_3OD) of compound **2**.

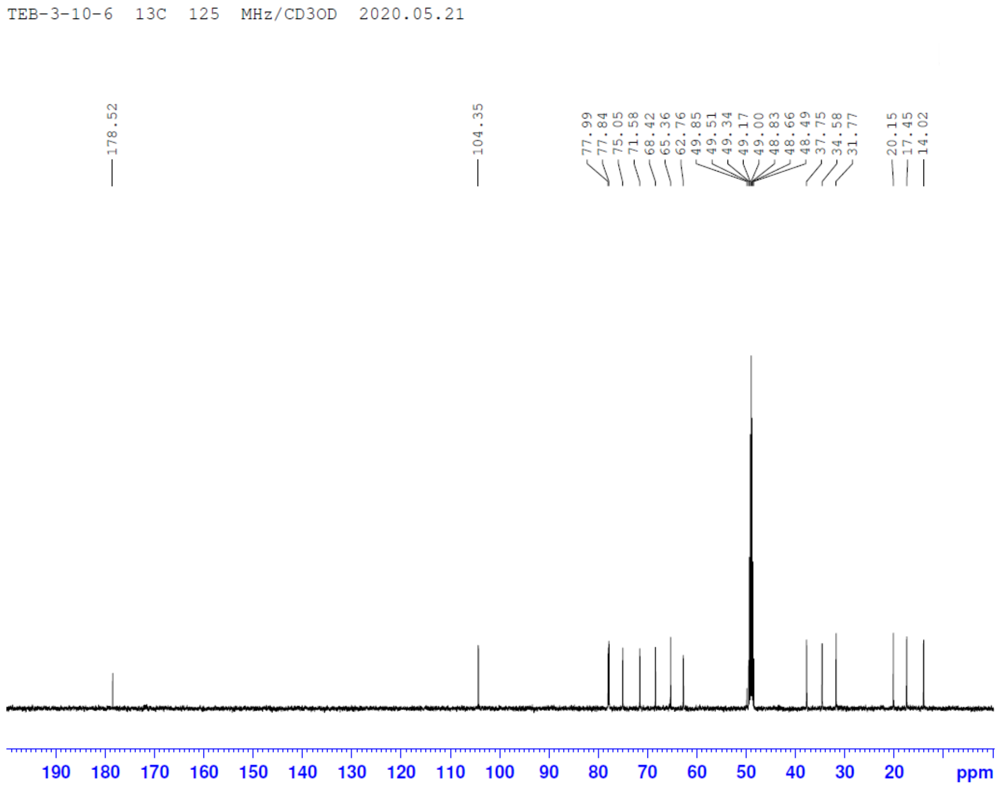

Figure S7. ^{13}C NMR (125 MHz, CD_3OD) of compound **2**.

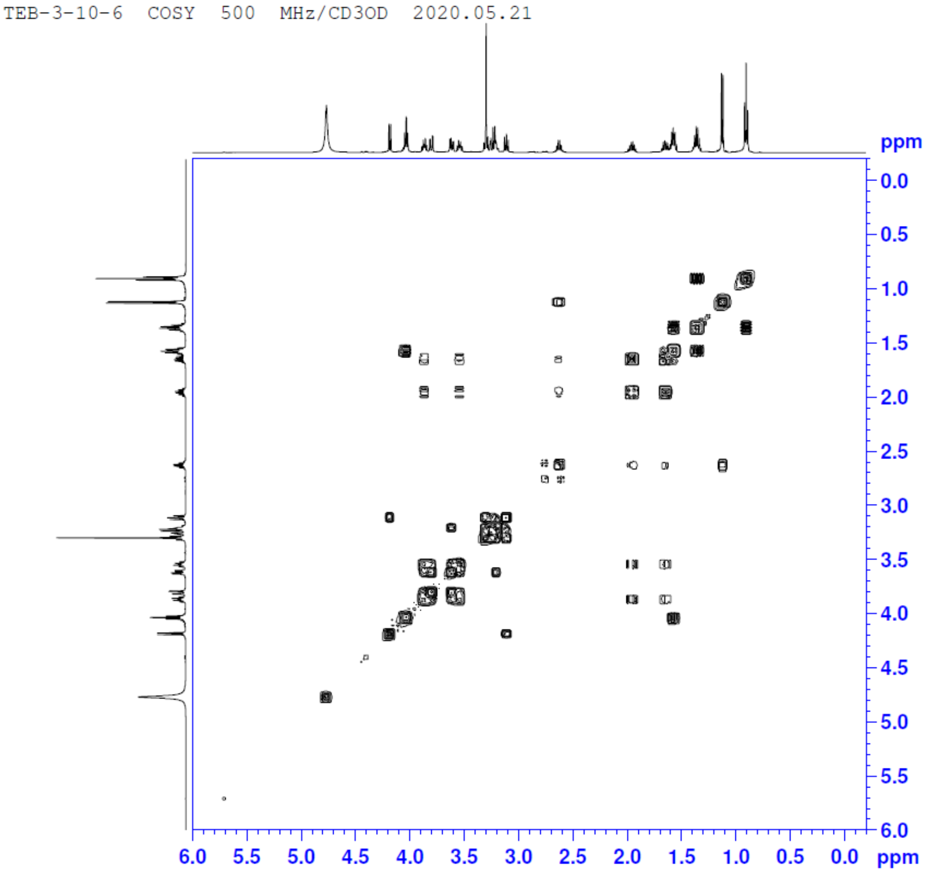

Figure S8. COSY spectrum (500 MHz, CD_3OD) of compound **2**.

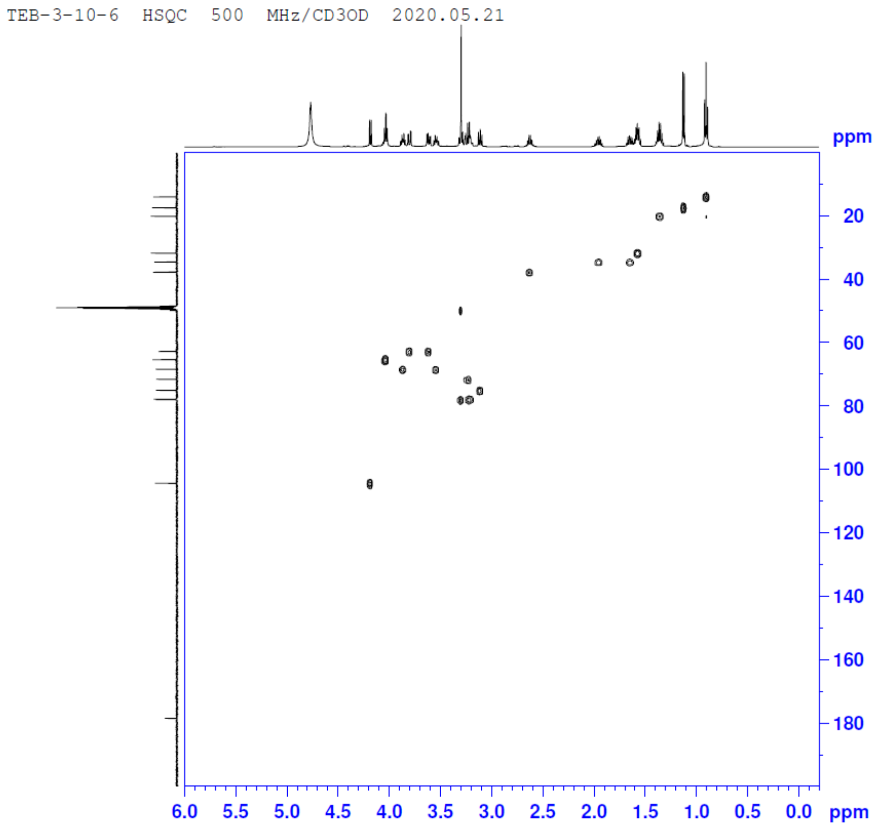

Figure S9. HSQC spectrum (500 MHz, CD_3OD) of compound **2**.

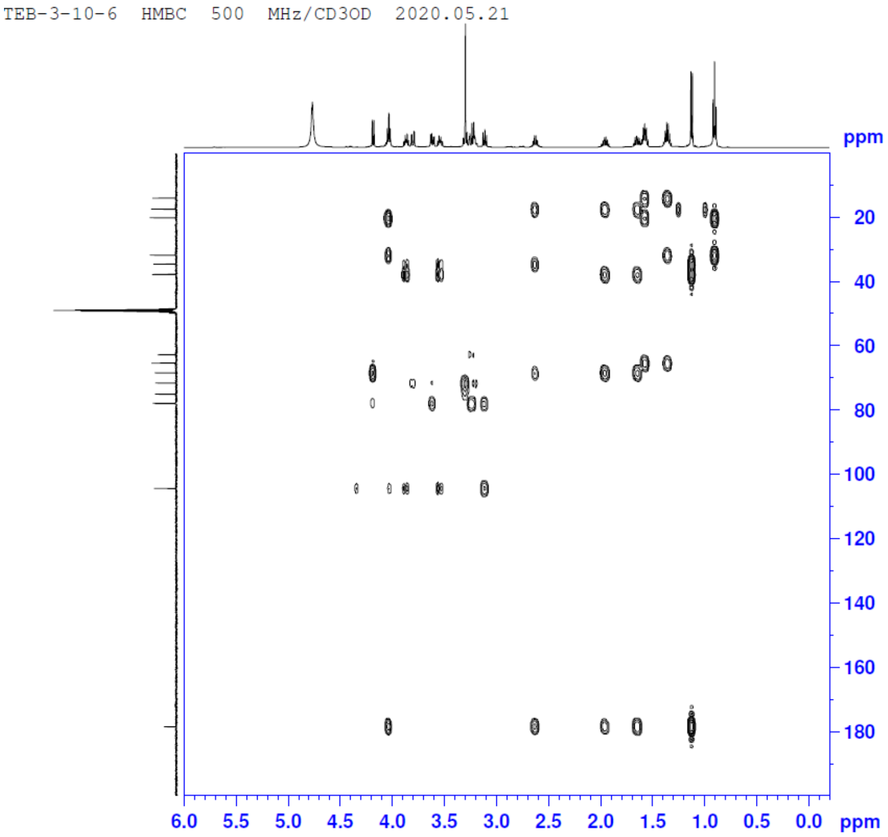

Figure S10. HMBC spectrum (500 MHz, CD_3OD) of compound **2**.

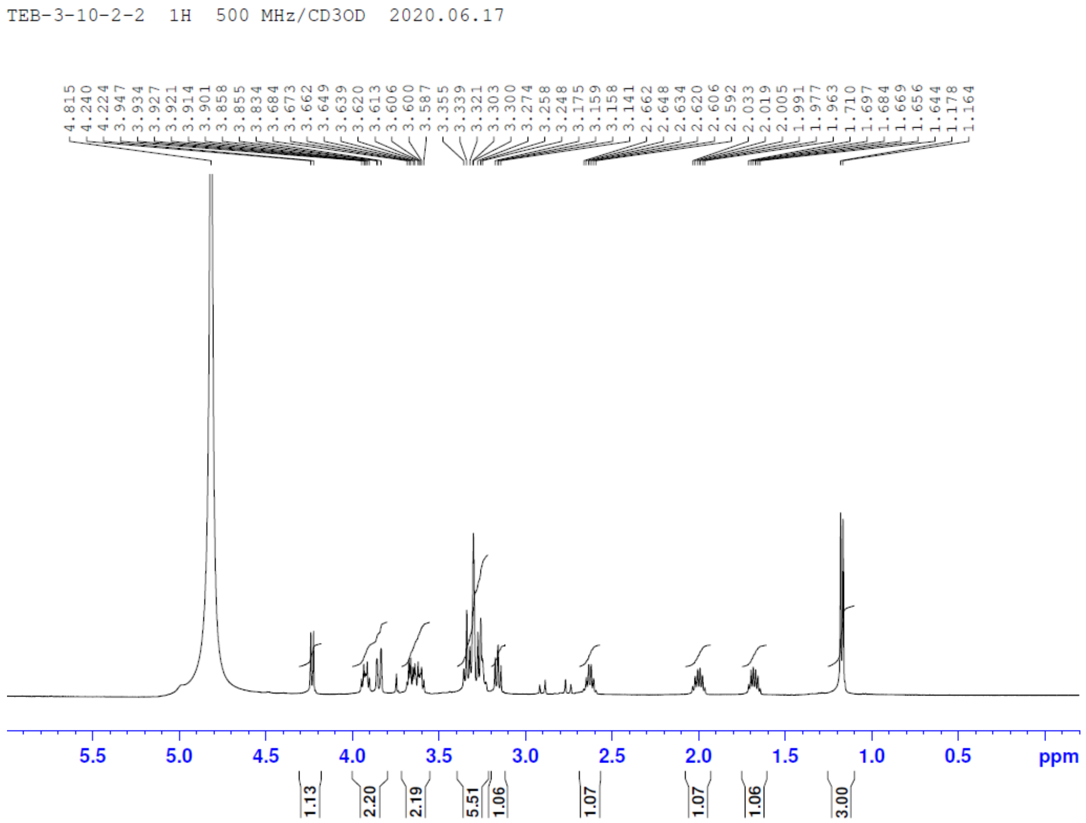

Figure S11. ^{1}H NMR (500 MHz, CD_3OD) of compound **3**.

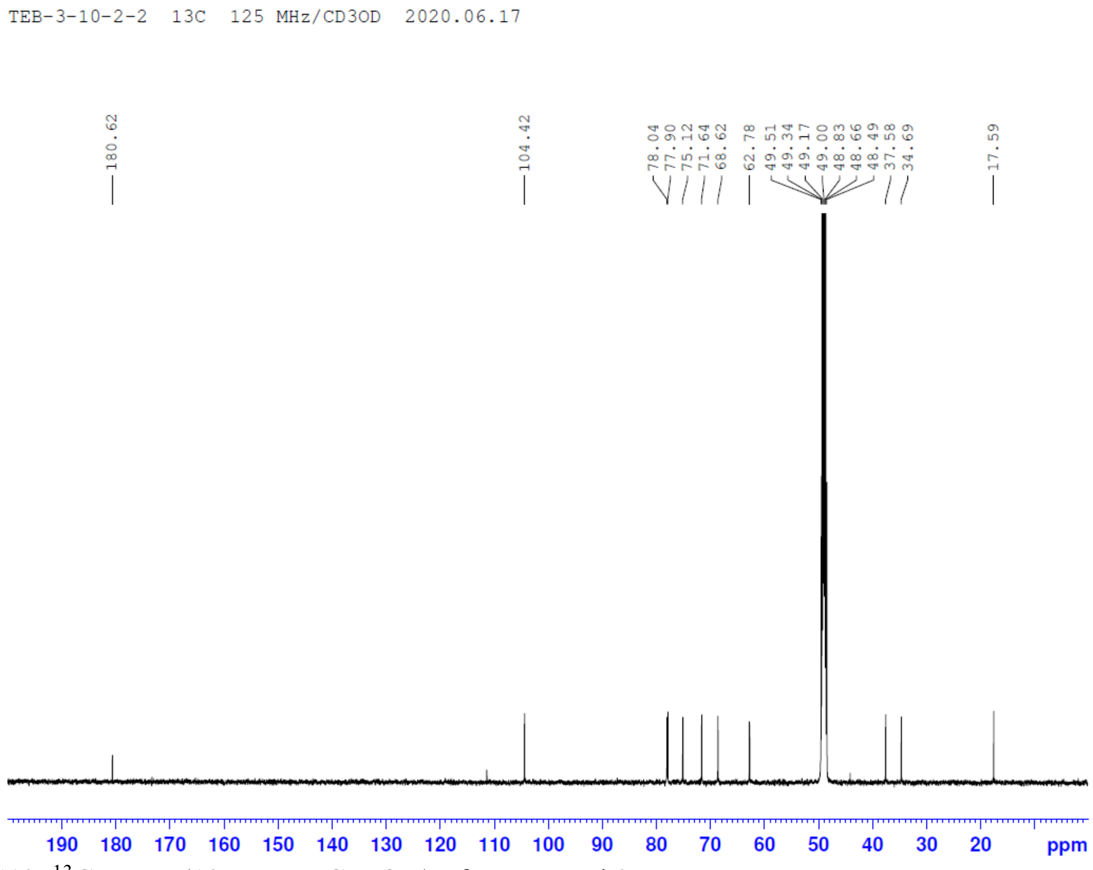

Figure S12. ^{13}C NMR (125 MHz, CD_3OD) of compound **3**.

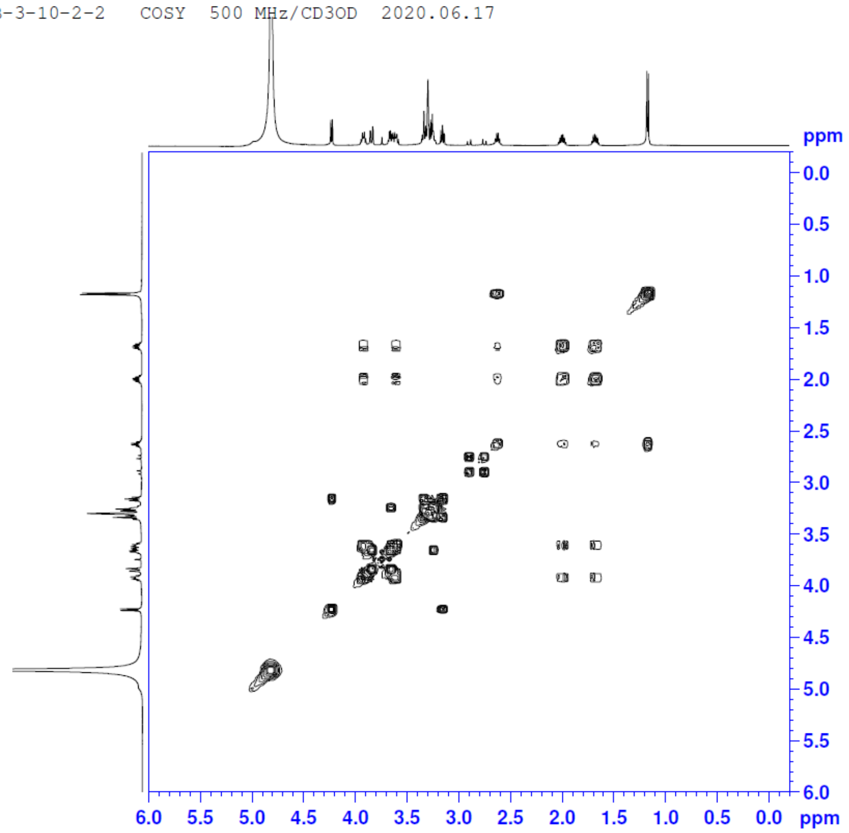

Figure S13. COSY spectrum (500 MHz, CD_3OD) of compound **3**.

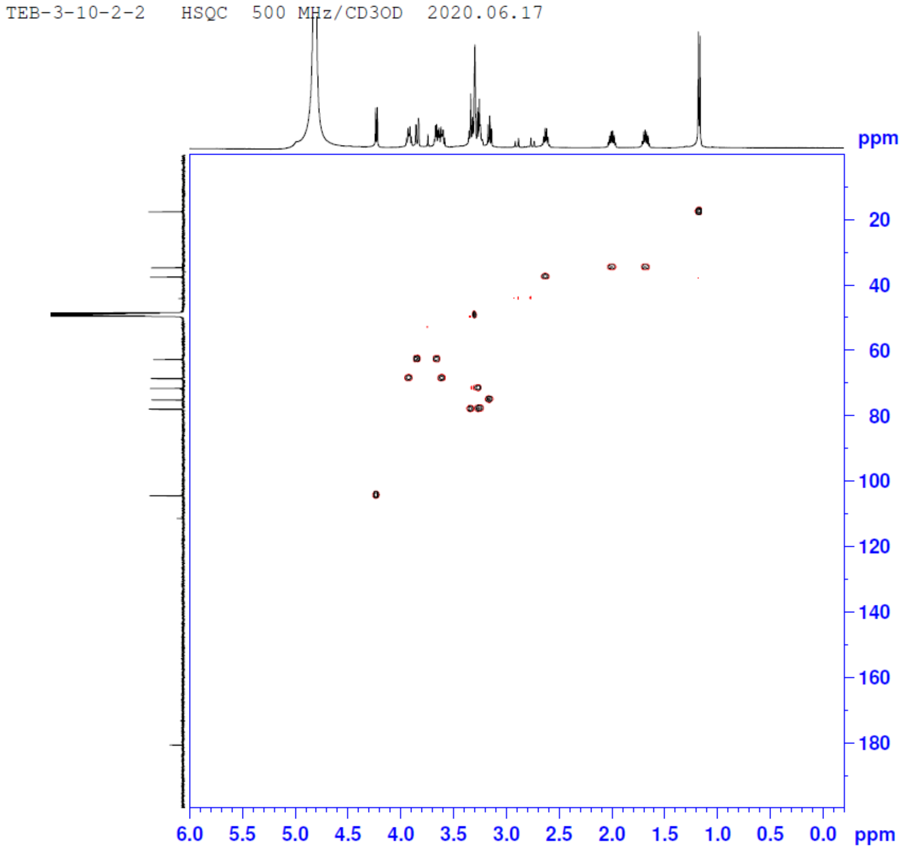

Figure S14. HSQC spectrum (500 MHz, CD_3OD) of compound **3**.

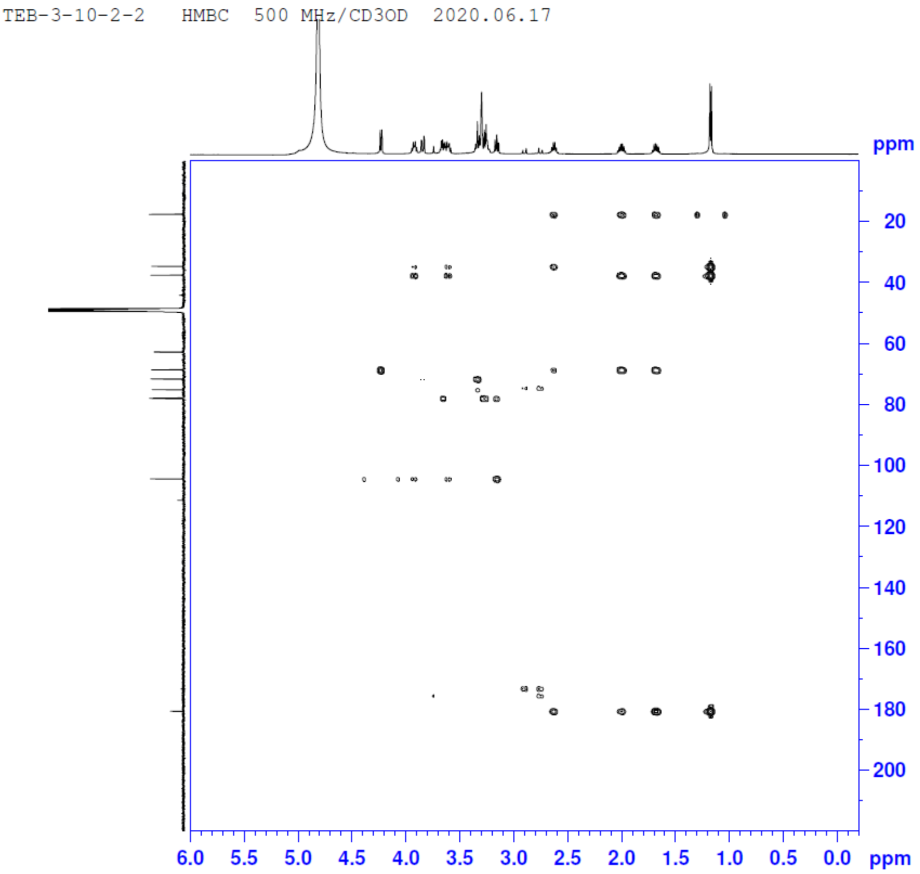

Figure S15. HMBC spectrum (500 MHz, C_5D_5N) of compound **3**.

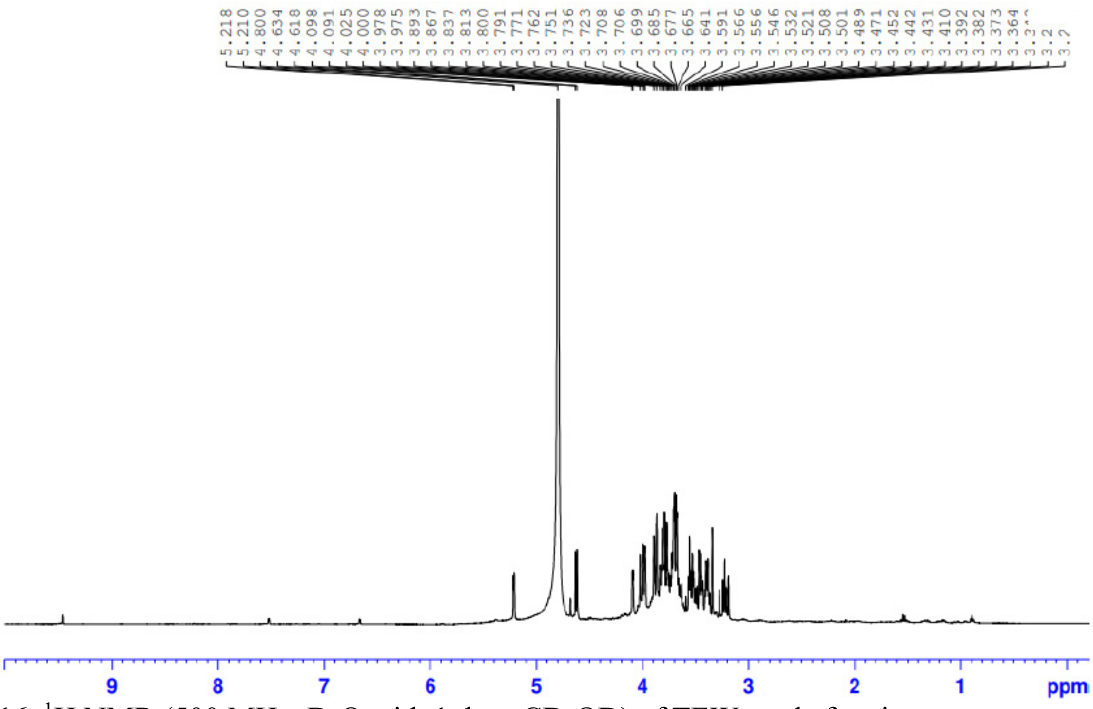

Figure S16. ^{1}H NMR (500 MHz, D_2O with 1 drop CD_3OD) of TEW crude fraction.

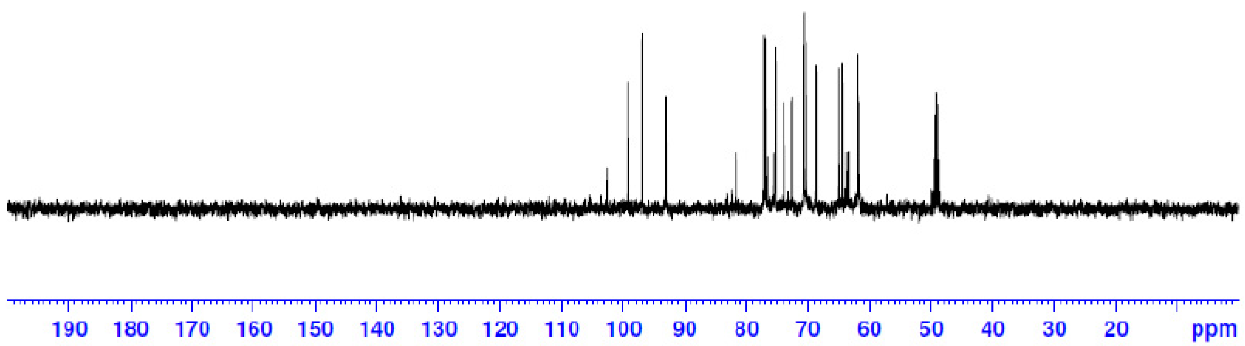

Figure S17. ^{13}C NMR (125 MHz, D_2O with 1 drop CD_3OD) of TEW crude fraction.

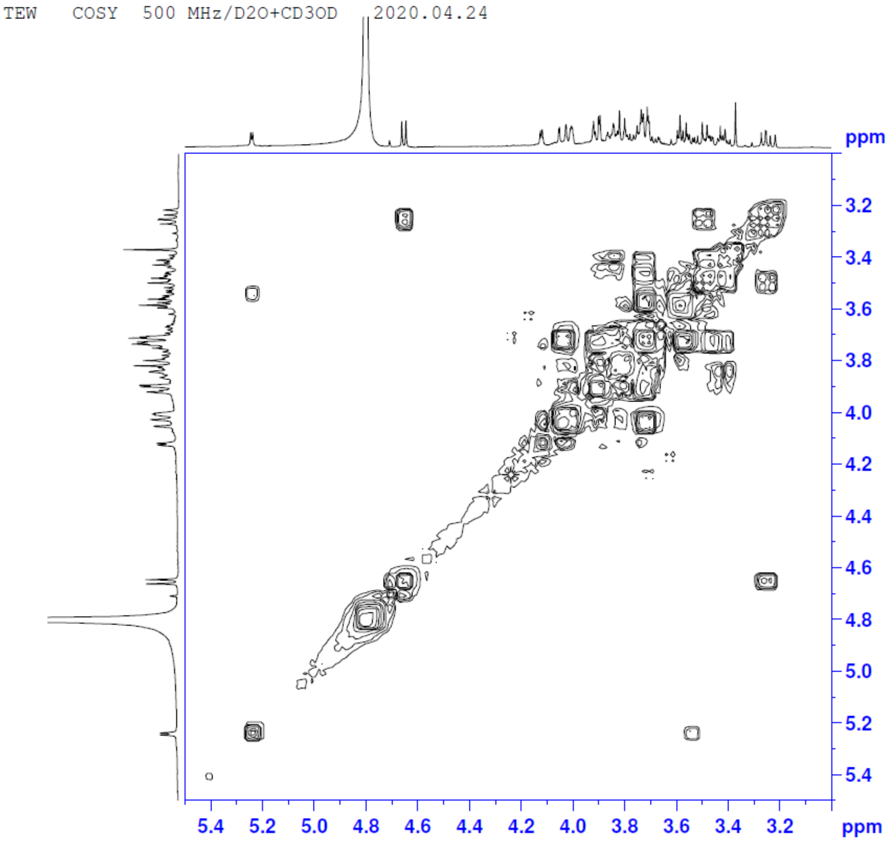

Figure S18. COSY spectrum (500 MHz, D_2O with 1 drop CD_3OD) of TEW crude fraction.

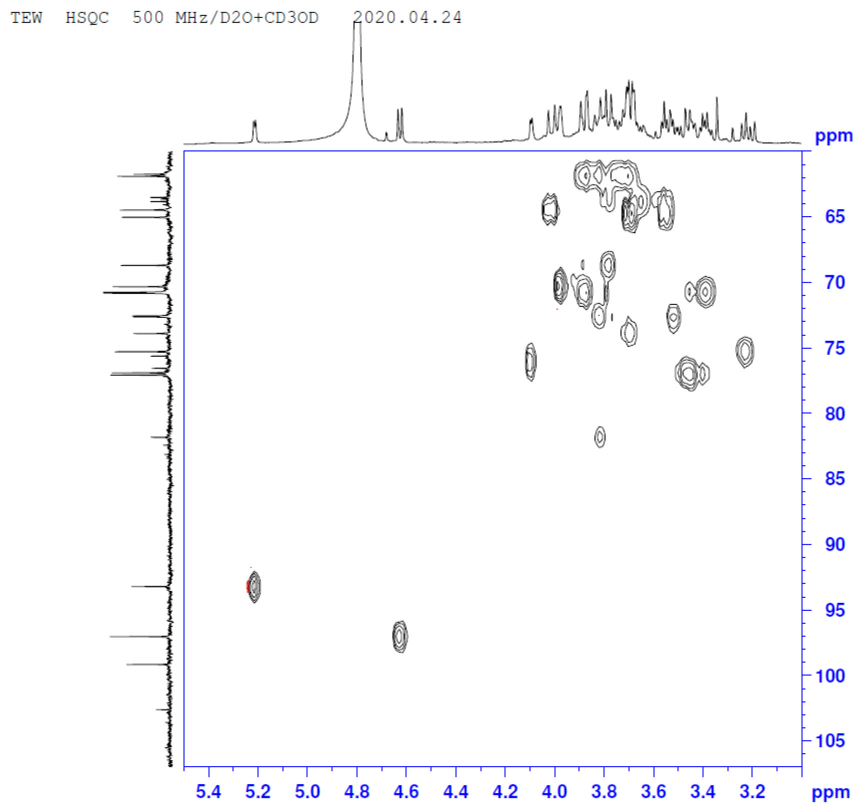

Figure S19. HSQC spectrum (500 MHz, D_2O with 1 drop CD_3OD) of TEW crude fraction.

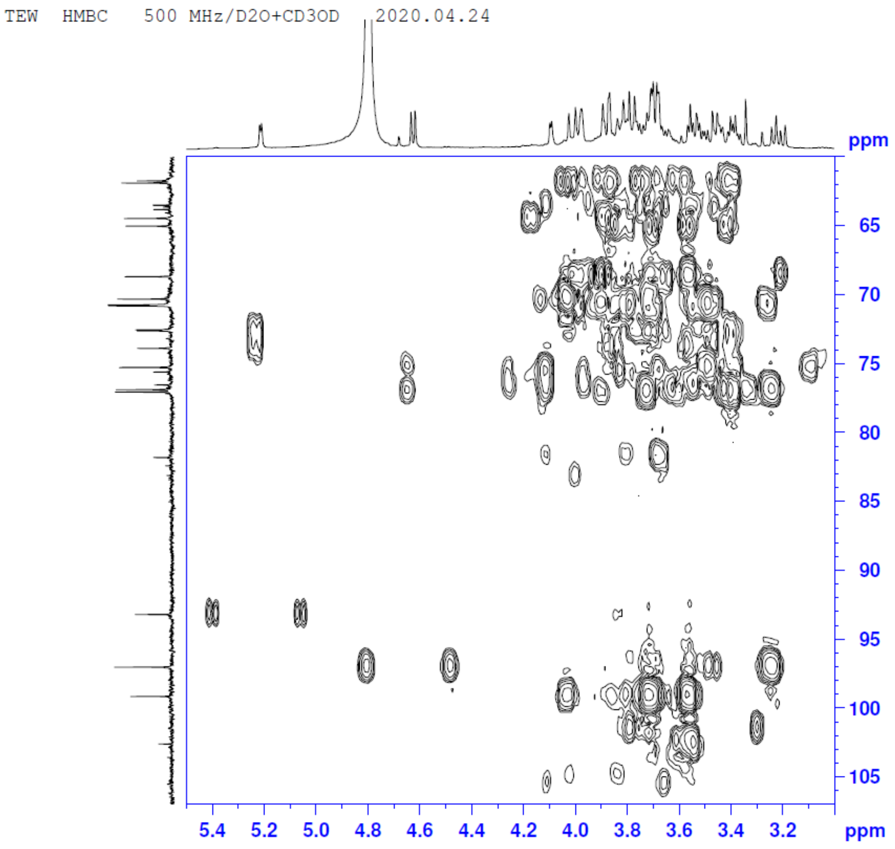

Figure S20. HMBC spectrum (500 MHz, D_2O with 1 drop CD_3OD) of TEW crude fraction.

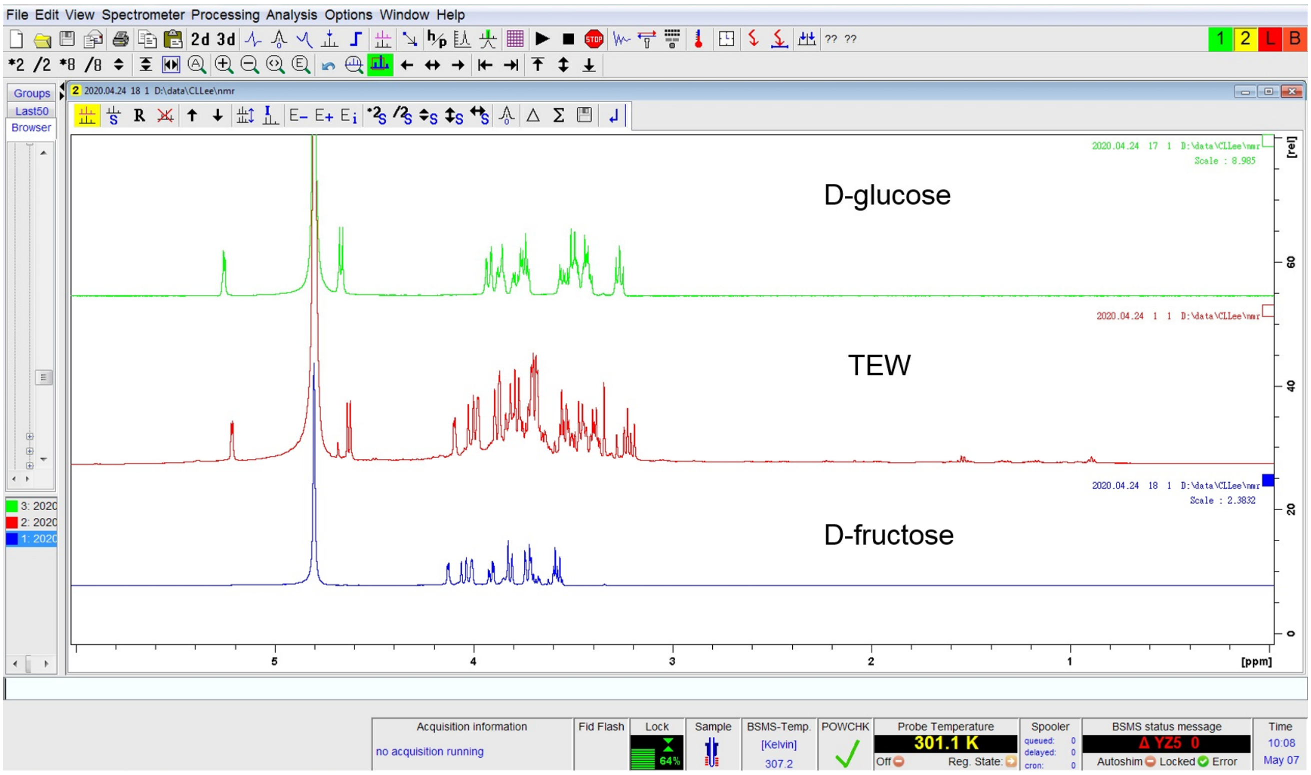

Figure S21. ^{1}H NMR (500 MHz, D_2O with 1 drop CD_3OD) of D-glucose standard, TEW, and D-fructose standard, respectively.

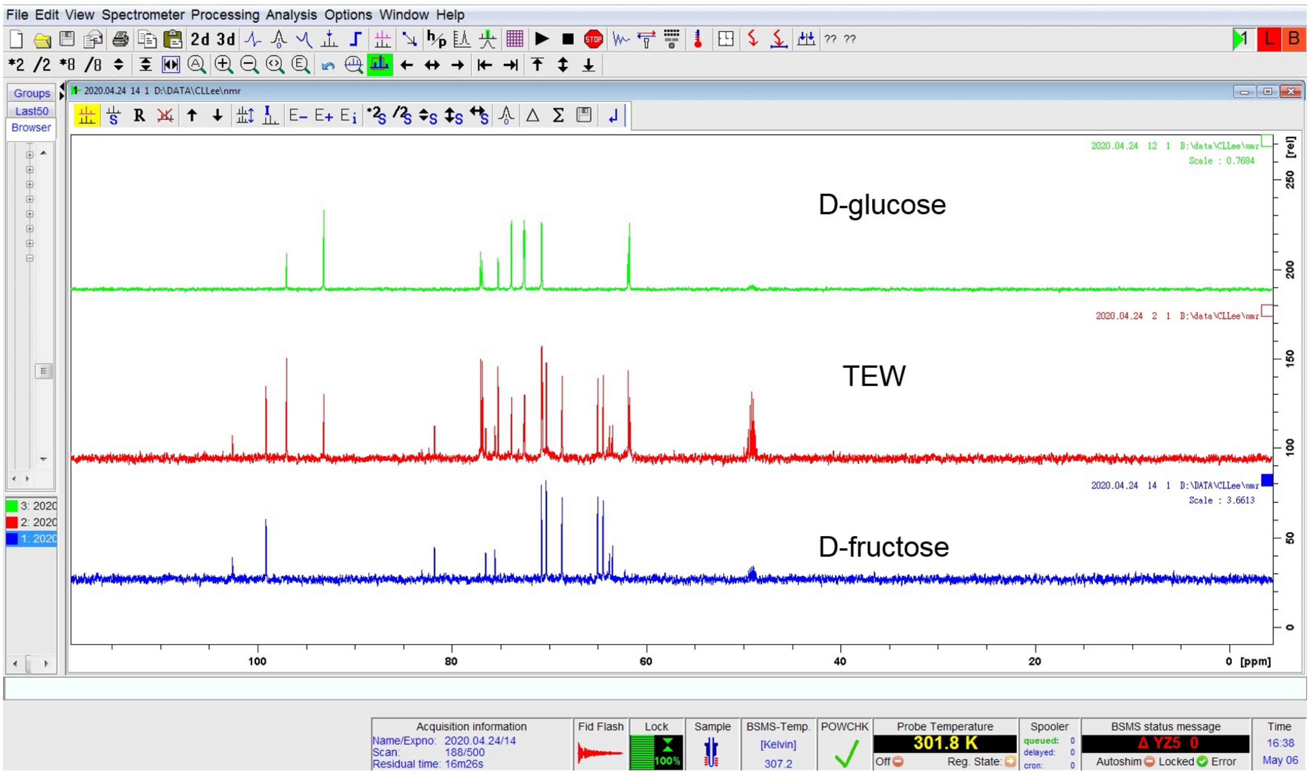

Figure S22. ^{13}C NMR (125 MHz, D_2O with 1 drop CD_3OD) of D-glucose standard, TEW, and D-fructose standard, respectively.

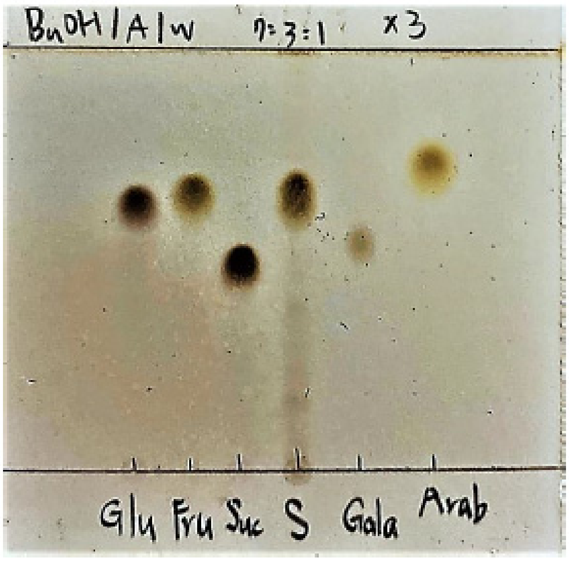

Figure S23. TLC analysis of TEW (S) were compared against glucose (Glc), fructose (Fruc), sucrose (Suc), galactose (Gala), and arabinose (Arab). Stationary phase: silica gel; Mobile phase: *n*-butanol/acetone/water, 7:3:1 for developing with three times. Being stained by spraying with 5% (v/v) sulfuric acid in MeOH solution before heating on a hot plate.

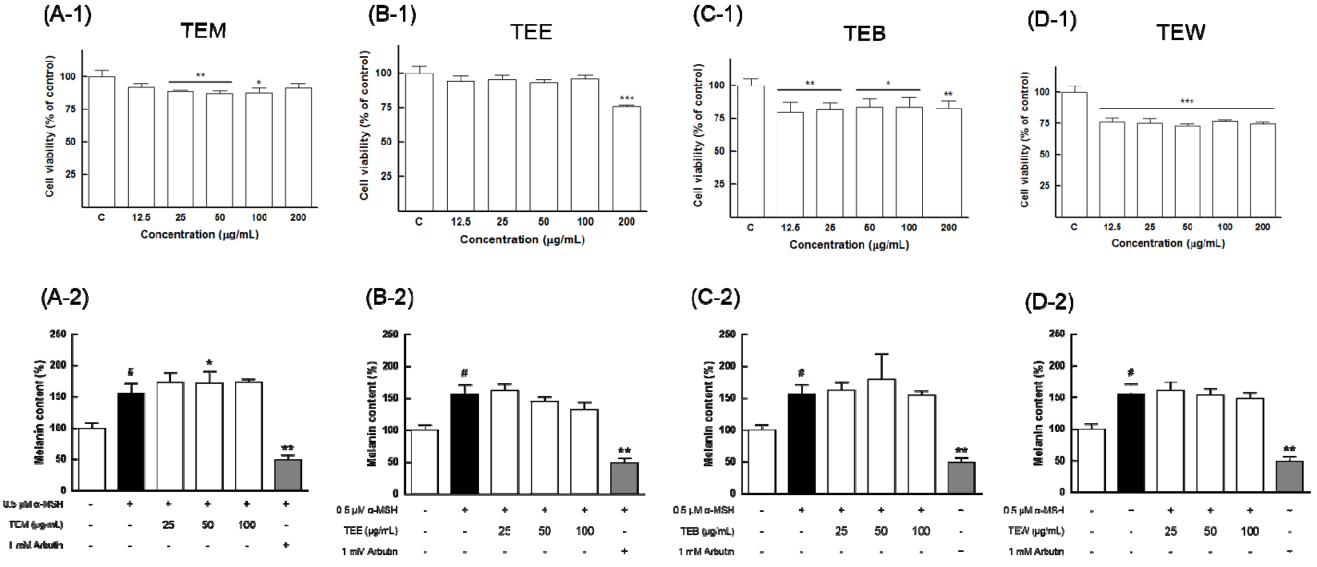

Figure S24. Cytotoxicity toward B16 (A-1 to D-1) and the regulation of melanogenesis effects in B16 cells (A-2 to D-2) of crude extract TEM, fractions TEE, TEB, and TEW, respectively. The data shown are representative of three independent experiments. #, $p < 0.001$ compared with control group, and *, $p < 0.05$, **, $p < 0.01$, and ***, $p < 0.001$ compared with the control group (A-1 to D-1) or α-MSH treated group (A-2 to D-2).

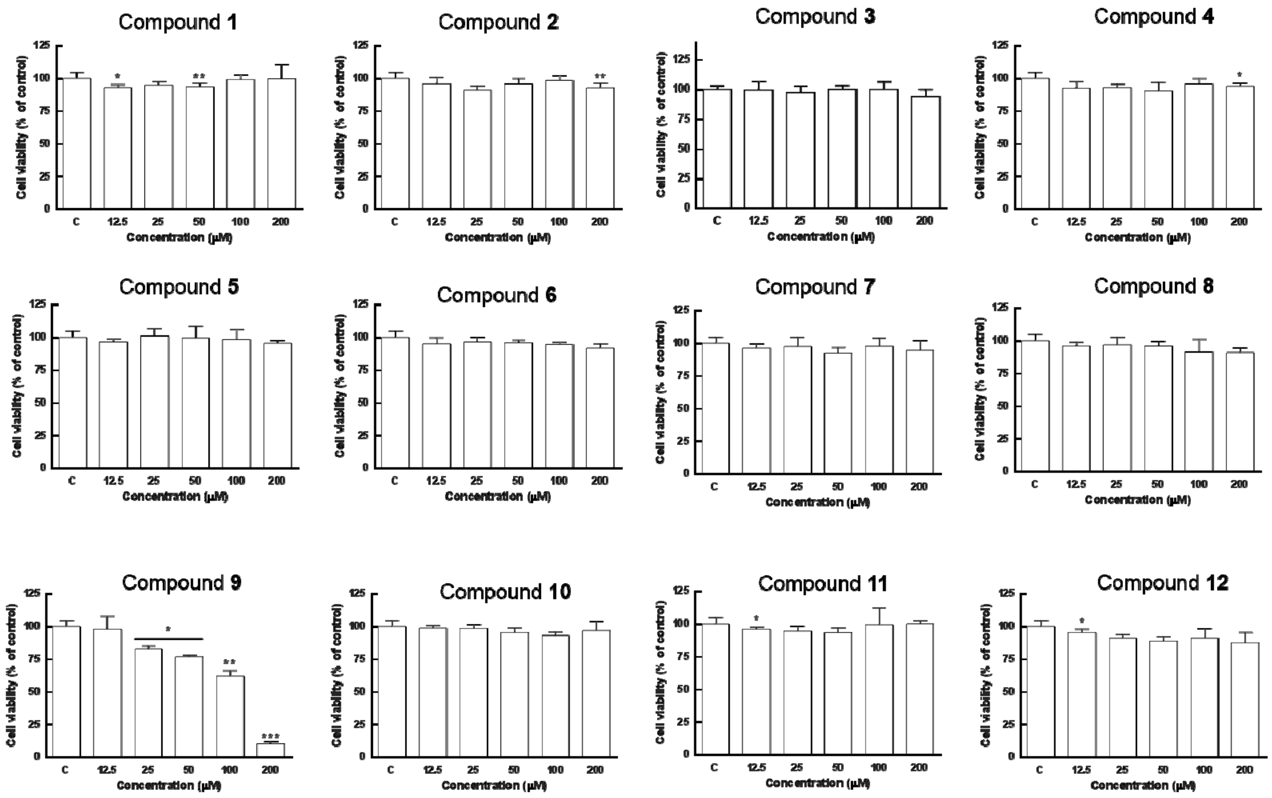

Figure S25. Cytotoxicity data of compounds **1**–**12** toward B16 cells. The data shown are representative of three independent experiments. *, $p < 0.05$, **, $p < 0.01$, and ***, $p < 0.001$ compared with the control group. Compound **9** has a cytotoxic IC_{50} value of 81.9 ± 1.3 μM.

Table S1. Tyrosinase activity effects of compounds **4**, **6**, **10**, and **12**.

Compd		Tyrosinase activity (%)	Compd		Tyrosinase activity (%)
	Control	100.0 ± 16.0		Control	100.0 ± 13.7
	α-MSH (0.5 μM)	254.3 ± 5.3		α-MSH (0.5 μM)	282.7 ± 19.2
	Arbutin (1 mM)	152.0 ± 14.2		Arbutin (1 mM)	145.2 ± 13.0
**4**	10 μM	290.1 ± 7.1	**6**	10 μM	327.5 ± 8.9
	20 μM	306.8 ± 16.5		20 μM	343.3 ± 17.6
	40 μM	309.9 ± 13.2		40 μM	344.5 ± 12.0
Compd		Tyrosinase activity (%)	Compd		Tyrosinase activity (%)
	Control	100.0 ± 13.3		Control	100.0 ± 13.7
	α-MSH (0.5 μM)	260.1 ± 10.2		α-MSH (0.5 μM)	305.5 ± 9.8
	Arbutin (1 mM)	140.2 ± 7.2		Arbutin (1 mM)	129.2 ± 12.4
**10**	10 μM	282.1 ± 13.2	**12**	10 μM	328.3 ± 11.6
	20 μM	288.6 ± 8.2		20 μM	339.3 ± 15.1
	40 μM	294.1 ± 9.2		40 μM	341.7 ± 14.9

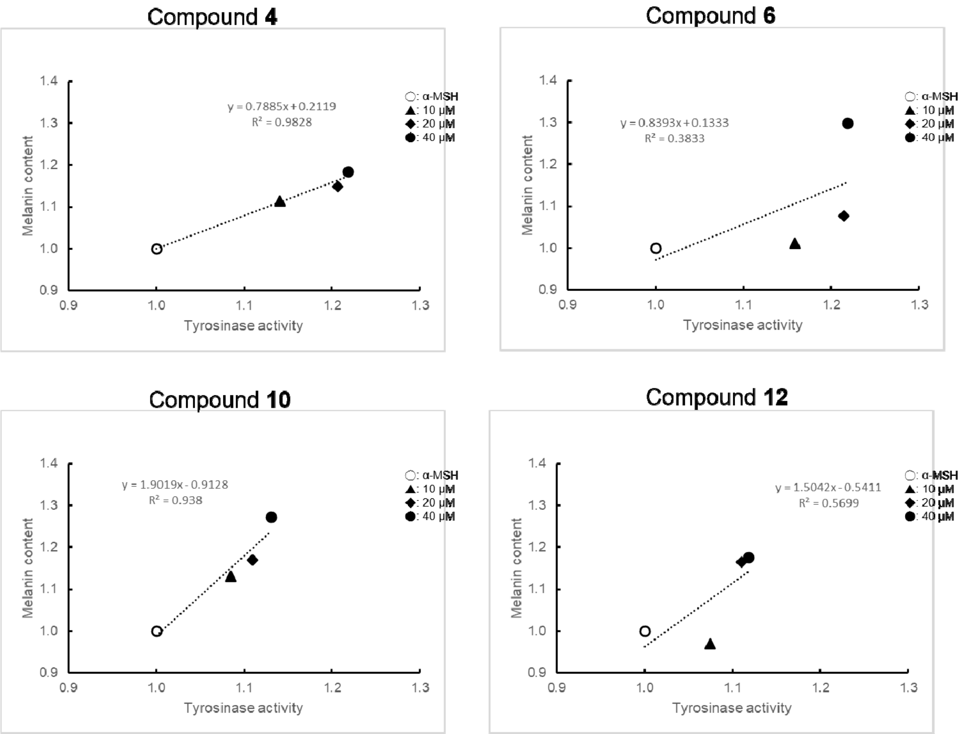

Figure S26. A correlation graph between the cellular melanin contents and the effects on tyrosinase of compounds **4**, **6**, **10**, and **12**.

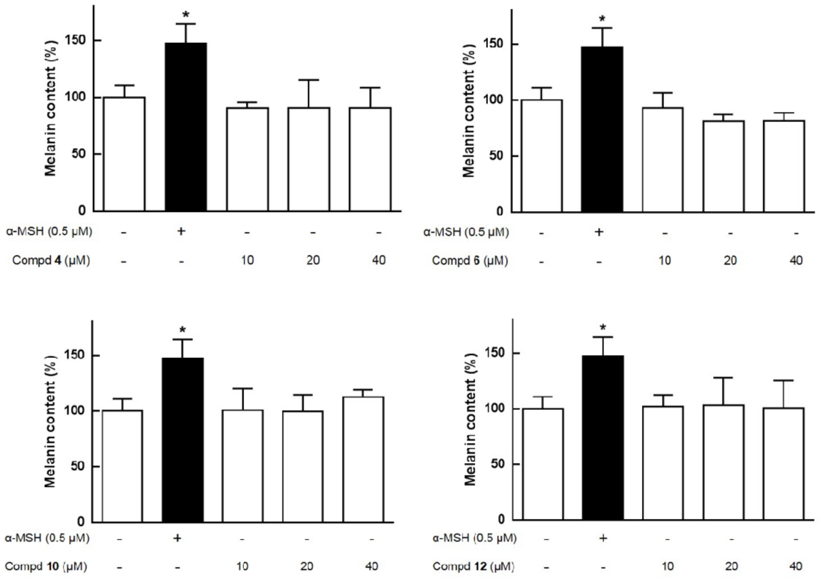

Figure S27. Effects of compounds **4**, **6**, **10**, and **12** without co-treated α-MSH on melanin content of B16 melanoma cells. *, $p < 0.05$ compared with control group.

(A)

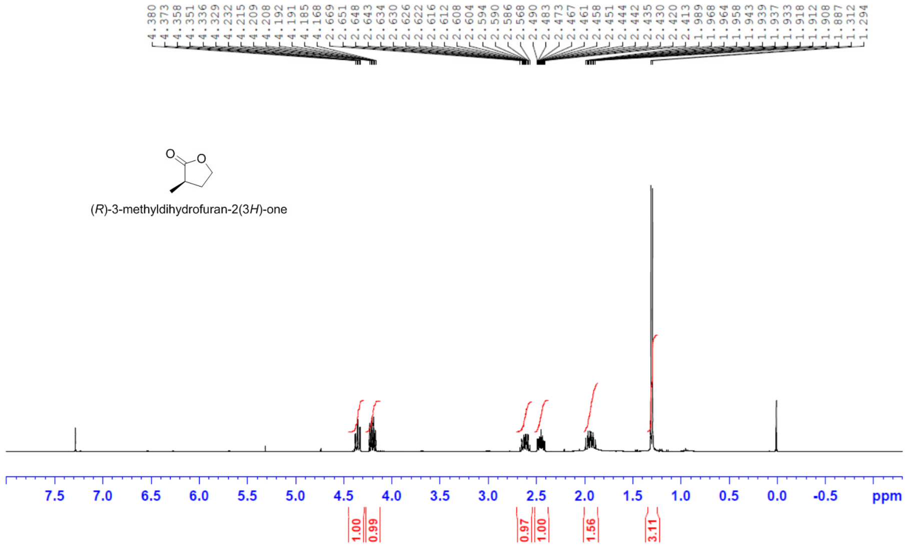

(B)

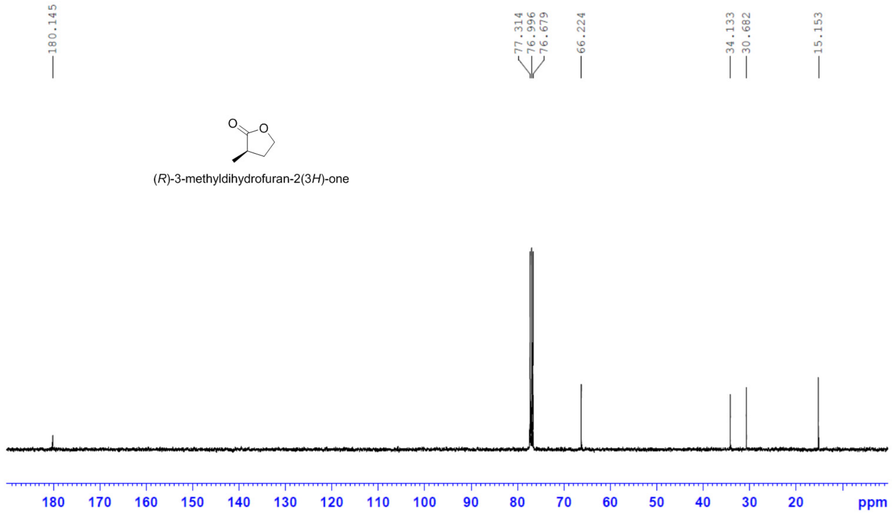

(C)

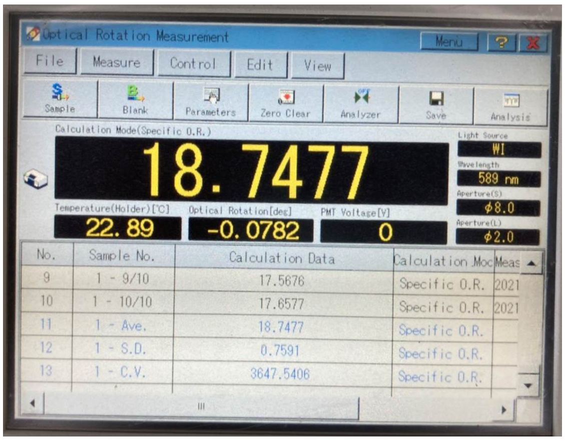

Figure S28. ^{1}H NMR (400 MHz, $CDCl_3$) (A), ^{13}C NMR (100 MHz, $CDCl_3$) (B), and optical rotation data (C) of the product, 3-methyldihydrofuran-2(3*H*)-one obtained from acid hydrolysis of compound **2**.

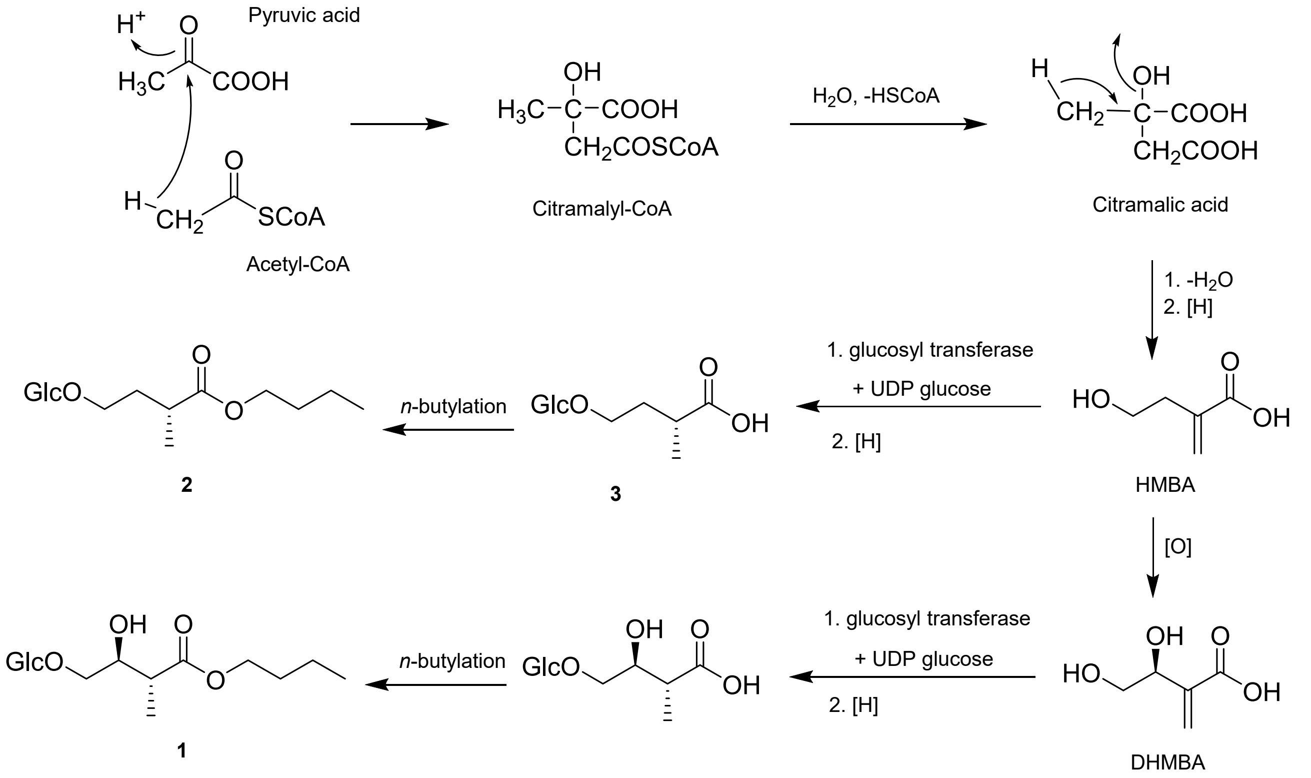

Figure S29. The possible biosynthesis of compounds **1**−**3** from D-glucose (Glc) and 4-hydroxy-2-methylenebutanoic acid (HMBA) and/or (*S*)-3,4-dihydroxy-2-methylenebutanoic acid (DHMBA). The UDP glucose and [H] mean uridine diphosphate glucose and chemical reduction, respectively.
